# Lipidomics combined with transcriptomic and mass spectrometry imaging analysis of the Asiatic toad (*Bufo gargarizans*) during metamorphosis and bufadienolide accumulation

**DOI:** 10.1186/s13020-022-00676-7

**Published:** 2022-11-04

**Authors:** Bo Sun, Shan Jiang, Mingli Li, Yan Zhang, Yanyan Zhou, Xiaolu Wei, Hongjie Wang, Nan Si, Baolin Bian, Haiyu Zhao

**Affiliations:** Institute of Chinese Materia Medica, China Academy of Chinese Medical Sciences, Beijing, 100700 China

**Keywords:** *Bufo gargarizans*, Metamorphosis, Bufadienolide, Lipidome, Transcriptome, Mass spectrometry imaging (MSI)

## Abstract

**Background:**

To adapt to life on land, Asiatic toads (*Bufo gargarizans*) must remodel their bodies and refine their chemical defenses in water. The full scope of the mechanisms underlying these processes has yet to be revealed. Bufadienolides (BDs) are chemical defense substances secreted by toads when they are in danger, and they have high medicinal value in treating heart failure, cancer, and hepatitis. However, the artificial breeding of toads to increase BDs has been unsuccessful due to the high mortality of toad larvae during metamorphosis.

**Method:**

Toad larvae at different growth stages were selected to study the changes in the metamorphosis process under the same growth conditions. The differences of tadpoles were explored, including body remodeling, energy metabolism, synthesis and regulation of BDs, through lipidomic technology, transcriptomic technology, and mass spectrometry imaging technology during metamorphosis.

**Results:**

During metamorphosis, tadpoles underwent significant changes in lipid metabolism due to body remodeling to adapt to terrestrial life, which involved ketosis, lipogenesis, cholesterol metabolism, and fatty acid oxidation. The accumulation trend of BDs was observed. “Pentose phosphate pathway” and “Aromatase activity” may be the critical pathway and GO term in BD synthesis, involving 16 genes predominantly expressed in the liver. The involved genes were mainly expressed in the liver, consistent with the synthetic site observed by mass spectrometry imaging.

**Conclusion:**

Together, our findings presented the changes in the toad larvae during metamorphosis and highlighted the accumulation process of BDs as well as the regulatory pathways and synthetic site, providing research and theoretical basis for future development of the toad resources.

**Supplementary Information:**

The online version contains supplementary material available at 10.1186/s13020-022-00676-7.

## Introduction

Toads are one of the most widely distributed amphibians. There are over 300 species in the world, of which the Asiatic toad (*Bufo gargarizans*) is an ecological generalist, widely distributed in East Asia, and has extremely high medicinal value [1]. Bufadienolides (BDs) enriched in the parotid venom glands of the toads have exhibited strong anti-tumor and anti-inflammatory activities [2, 3]. Chansu (unrefined BDs) is a traditional Chinese animal-derived Chinese medicine in China. However, with the continuous advancement of urbanization in various places, and the increasing environmental degradation, the number of these toads has gradually decreased [4, 5]. To protect species diversity, the Chinese government included *B. gargarizans* on the protected animals list called “Three Animals.” Chansu also was included on the list of prohibited export commodities, which reflects the scarcity of toads and BDs. As early as the 1960s, the chemical synthesis of BDs had been attempted, but it failed to be applied to industrial production [6, 7].

Obviously, artificial breeding and biosynthesis are shortcuts that will solve the current contradiction between resource conservation and utilization. The implementation is easy for obtaining high-purity medicinal bufadienolides. However, there are considerable obstacles in this solution pathway because of the high mortality of toads during the breeding process. The toads are susceptible to external interference during metamorphosis [8–10]. In addition, the regulatory genes governing BD biosynthesis remain unclear, and the enzymatic metabolic pathways and transformational relationships between BDs and synthetic precursors cannot be elucidated.

BDs are important chemical defense substances produced during the growth process of toads and already exist in toad eggs [11, 12]. Parotid venom glands for secreting and storing BDs begin to develop after landing. It is possible that the BDs produced will gradually become more abundant with the growth and development of *B. gargarizans*. However, the body fat of toads will be gradually decreasing. On the one hand, the tadpoles eat less during the metamorphosis and consume a lot of lipids to maintain energy supply [13]; on the other hand, cholesterol is the synthetic precursor of BDs, and the decrease in lipids may also be related to this. Therefore, conducting a correlation study between lipidomics and transcriptomics of toad larvae at different growth stages is very important. Thus far, there have been few omics studies on the growth and development of *B. gargarizans*, mainly because the basic information of toads collected in the wild was unclear, such as age, living environment, and genetic background. To ensure high conservation of the samples, we started hatching from the egg belt and tested tadpoles or toads at different stages. Thus, the results will reflect the differences in BDs, lipids, and gene expression at different growth stages.

Transcriptomics and lipidomics are potent tools for biological research because they can accurately reflect the changes in the organism and reveal the relationship between lipids and physiological processes from a unique perspective [14–19]. Xu et al. revealed the significant changes in the composition of glycerolipids, glyphospholipids, and sphingolipids in the response of mammalian white adipose tissue to short-term cold exposure, as well as the potential gene regulation process through the conjoint analysis of lipidome and transcriptome [20]. In addition, mass spectrometry imaging technology is a new molecular method that can directly obtain information such as molecular mass, relative abundance, and tissue distribution of small molecular substances in animals from the surface of tissue slices and display them in the form of color images. It is a method widely used to study the distribution of drugs and their metabolites in the body [21–23].

The following research was carried out to provide a scientific and technological basis for artificial breeding and biosynthesis and clarify the changes in the body and BDs during metamorphosis. The pre-metamorphosis (Gosner stage 31 and 38), metamorphosis climax (Gosner stage 42), and metamorphosis complete (Gosner stage 46) of *B. gargarizans* were selected from the same egg mass for examination. Ultra-high performance liquid chromatography-tandem mass spectrometry (UPLC-MS/MS) was used to conduct comprehensive lipidomic analysis, identification, and semi-quantitative analysis of differential lipids and BDs, to reveal the lipid changes in body composition and accumulation of BDs. Combined with transcriptomic analysis, the key regulatory pathways and genes associated with lipids and BDs during metamorphosis were analyzed. Furthermore, desorption electrospray ionization mass spectrometry imaging (DESI-MSI) was used to study the synthesis position of BDs, and the important role of the liver in BD biosynthesis was determined. The results of this study will provide a theoretical basis for metamorphosis research, BD biosynthesis, and artificial breeding of *B. gargarizans*.

## Materials and methods

### Animal materials

**Fig. 1:**
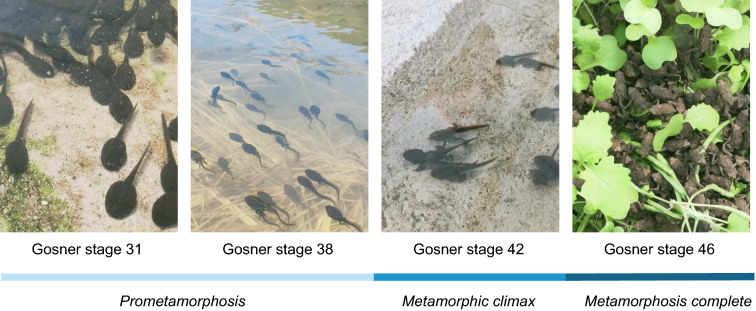
The four life stages of *Bufo gargarizans*

To reduce the influence of age and environment on sample quality, tadpole eggs from one egg mass were collected in Sikai Town, Liangshan Yi Autonomous Prefecture, Sichuan Province, China. Tadpole eggs were hatched by Xichang Fuhua Jinchan Bio-Tech Co., Ltd. (Sichuan, China). After hatching, these tadpoles were placed in a 4.0 m by 10.0 m by 0.7 m outdoor rearing pond. The temperature of the outdoor rearing tank was maintained between 13 and 17 °C, and the humidity was greater than 70%. The developmental stages of tadpoles were identified according to Gosner, and four stages in metamorphosis were detected. Namely, G31 (Gosner stage 31, pre-metamorphosis), G38 (Gosner stage 38, pre-metamorphosis), G42 (Gosner stage 42, metamorphosis climax), and G46 (Gosner stage 46), metamorphosis complete), as shown in Fig. 1. All of the tadpoles were grouped according to their stage and euthanized by flash freezing in dry ice after fasting for 12 h.

### Sample processing

With four tadpoles as one sample, three biological replicates were applied to the later transcriptomic analysis and six biological replicates for lipidomic analysis. The samples were ground into powder using liquid nitrogen and mixed evenly for subsequent analysis. Precisely, 100 mg sample was weighed and put into a centrifugal tube containing 1 mL methanol. After that, the filled centrifugal tubes were vortexed for 30 s and sonicated for 30 min in an ice-cold sonication bath. The suspension was clarified by centrifugation at 12,000 rpm for 10 min at 4 ℃. The 700 µL of supernatant was moved to a clean centrifuge tube, dried under a nitrogen blow dryer, and then dissolved in 150 µL methanol. Exactly 10 μL of each treated sample was taken and mixed into the QC sample for testing on the machine. All reagents used were chromatographically pure.

### Chromatographic conditions

Chromatographic separation was performed on a Thermo Ultimate 3000 hyperbaric liquid chromatography (LC) system. The chromatography column used was an Accucore C18 (2.6 μM, 2.1 × 150 mm) purchased from ThermoFisher Scientific. Positive/negative ion mode was used. The mobile phases were 0.1% formic acid in water (A) and acetonitrile (B). The gradient program used was as follows: 0–1 min, 25–40% B; 1–2 min, 25–40% B; 2–12 min, 40–60% B; and 12–22 min, 60–100% B. The flow rate was 0.4 mL/min, injection volume was 10 µL, and the column temperature was 35 °C. The sample was placed in a 4 °C automatic sampler for analysis.

### Mass spectrometry conditions

Primary and secondary mass spectrometric data were collected under software (Xcalibur V3.0, ThermoFisher Scientific, San Jose, CA, USA)-controlled Base Peak mode using a hybrid linear ion trap Orbitrap Velos mass spectrometer (LTQ-Orbitrap Velos, Thermo). The ESI source parameters were as follows: ion spray voltage, 3.4 kV; sheath gas flow rate, 35 arb; aux gas flow rate, 10 arb; capillary temperature, 350 °C; and S-lens RF level, 60%. The mass resolution of Fourier transform (FT) was 30,000 with a full scan in the range of *m*/*z* 100–1200. The MS/MS experiments were set as data-dependent scans. Data processing was performed using Xcalibur 3.0 and Chromeleon Xpress (version 7.2; ThermoFisher Scientific, CA, USA).

### Lipidomic data processing and statistical analysis

For non-targeted lipidomics, all the data obtained were processed using Progenesis QI software for imputing raw data, aligning and selecting peaks, and normalization to produce peak intensities for retention time (tR) and *m*/*z* data pairs. The resulting data were exported to SIMCA software (version 14.1.0, Sweden) for principal component analysis (PCA) and orthogonal partial least-squares discrimination analysis (OPLS-DA). Identification was performed using Progenesis QI (version 2.4, Waters, USA) software’s online Lipid Maps Structure Database (LMSD) database, Kyoto Encyclopedia of Genes and Genomes (KEGG) database, and Human Metabolome Database (HMDB) database, and theoretical fragment recognition was conducted. The precursor ion mass number deviation was 10 ppm in each case, and the fragment ion mass number deviation was 5 ppm in each case. Relevant statistical analysis of BDs was performed using GraphPad Prism 8.0 (GraphPad Software, USA).

### Sequencing of mRNA and data processing

RNA sequencing (RNA-seq) analysis was outsourced to OE Biotech (Shanghai, China). Briefly, total RNA was extracted using the mirVana miRNA Isolation Kit (Ambion, USA) following the manufacturer’s protocol. RNA integrity was evaluated using the Agilent 2100 Bioanalyzer (Agilent Technologies, USA). The untreated samples with RNA Integrity Number (RIN) ≥ 7 were subjected to the subsequent analysis. Libraries were constructed using a TruSeq Stranded mRNA LTSample Prep Kit (Illumina, USA) according to the manufacturer’s instructions. Then, these libraries were sequenced on the Illumina NovaSeq6000, and 125 bp/150 bp paired-end sequences were generated.

The clean sequences were mapped to the *B. gargarizans* genome (ASM1485885v1) using HISAT2. FPKM of each gene was calculated using Cufflinks, and the sequence counts for each gene were obtained by HTSeqcount. Differential expression analysis was performed using the DESeq2 (2012) R package. P-value < 0.05 and fold-change > 2 or fold-change < 0.5 was set as the threshold for differential expression genes (DEGs). GO enrichment (http://www.geneontology.org/) and KEGG pathway analysis (https://www.genome.jp/kegg/) of DEGs were performed to demonstrate the significantly enriched GO terms and metabolic pathways of DEGs in different groups and samples. All of the charts (bar charts, boxplots, Venn diagrams, bubble charts, and heat maps) were drawn using Oebiotech tools, a free online platform for data analysis (https://cloud.oebiotech.cn/task).

### RT-qPCR

RNA was extracted from tadpoles of the four stages and used for RT-qPCR. The primer sequences were designed in the laboratory and synthesized by TsingKe Biotech based on the mRNA sequences obtained from the NCBI database (Additional file 1: Table S1). Three biological repeats and three technical repeats were performed. Quantification was performed by a two-step reaction process: RT (reverse transcription) and PCR. Each RT reaction consisted of 0.5 μg RNA, 0.5 μL of gDNA Remover, and 2 μL of 5 × TransScript All-in-One SuperMix for qPCR, in a total volume of 10 μL. Reactions were performed using the GeneAmp® PCR System 9700 (Applied Biosystems, USA) for 15 min at 42 °C, and 5 s at 85 °C. A 10 × dilution in nuclease-free water was performed for the 10 μL RT reaction mix, which was then stored at − 20 °C.

Real-time PCR was performed using a LightCycler® 480 II Real-time PCR Instrument (Roche, Swiss) with 10 μL PCR reaction mixture that included 1 μL of cDNA, 0.2 μL of forward primer, 5 μL of 2 × *PerfectStart*™ Green qPCR SuperMix, 3.6 μL of nuclease-free water, and 0.2 μL of reverse primer. Reactions were incubated in a 384-well optical plate (Roche, Swiss) at 94 °C for 30 s, followed by 45 cycles of 94 °C for 5 s, and 60 °C for 30 s. The expression levels of mRNAs were normalized to ACTB and were calculated using the 2^−ΔΔCt^ method [24].

### DESI-MSI

The toads at the G46 stage were frozen to death with dry ice, and then their limbs were excised and embedded in 10% gelatin until they could be demolded. Whole-body cryosections were obtained using a Leica CM 3500 (Leica Microsystems, Nussloch, Germany) microtome at 100-μm increments. Each section was adhered to a positively charged coated slide (Citotest, Haimen, China). Serial tissue sections for optical microscopy and DESI-MSI were alternately collected to allow correlation and organ identification.

Mass spectrometric data were collected under software (HDImaging V1.4, Waters, Milford, MA, USA)-controlled Base Peak mode using a mass analyzer Xevo G2-XS QToF mass spectrometer (Waters, Milford, MA, USA). The DESI source parameters were as follows: collision voltage, off; spatial resolution, 200 μm × 200 μm; scan rate, 500 μm/s; spray solvent, 95% methanol in water with 200 pg/μL LE; spraying rate, 5 μL/min; gas nebulizer (N_2_) pressure, 0.5 Mpa; spray angle, 60°. The mass analysis mode of the Xevo G2 XS GToF was sensitivity mode with a full scan in the range of *m*/*z* 300–500. The ion mode was positive, and the instrument was tuned to a signal strength above 1e^6^ using a red Sharpie pen (rhodamine 6G, *m*/*z* 443). Data processing was performed using HDImaging 1.4. To observe the parotid gland and additional tissues and organs, three regions of the body in the left sagittal, mid-parasagittal, and horizontal planes were analyzed [25, 26]

## Results

### Significant changes in the lipid composition of *B. gargarizans* during metamorphosis

**Fig. 2:**
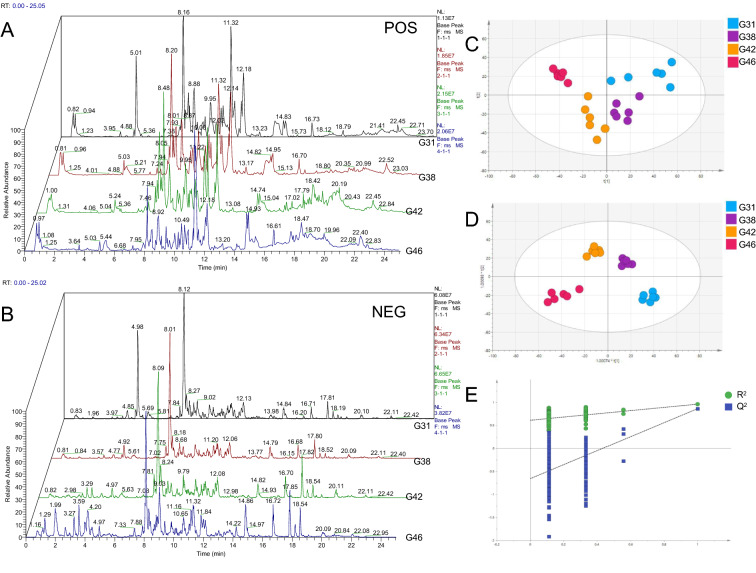
Mass spectrum of *B. gargarizan*s at different stages. BPI (base peak intensity chromatogram) of positive and negative ions [**A**: positive ion; **B**: negative ion]. Multivariate statistical analysis of lipid molecules [**C**: PCA score chart; **D**: OPLS-DA score chart; **E**: model validation diagram for OPLS-DA]

To ensure high-quality data acquisition, the performance of the LC–MS platform was monitored before and after analyzing each block based on QC samples. The samples were subjected to qualitative analysis, as shown in Fig. 2A and B. The base peak chromatograms (BPI) of samples at four stages were overlapped and compared. The results showed that the chromatographic peak response intensity and retention time of different samples partially overlapped, indicating that the four groups of samples were comparable.

The unsupervised principal component analysis (PCA) method was applied to obtain analysis results that were more intuitive and visual for the different developmental stages. The gathered results indicated that the samples for each stage could be effectively classified and characterized, and the groups grew further apart as the tadpoles developed. QC samples clustered together displayed satisfactory data quality (Fig. 2C). Orthogonal partial least squares-discriminant analysis (OPLS-DA) was used for variables with significant changes in the micromolecules among each group. The parameters of the OPLS-DA score plot obtained from these four groups (Fig. 2D) were R^2^X = 0.817, R^2^Y = 0.967, and Q^2^ = 0.805, which indicated excellent fitness and reliability. The model was validated by the permutation test (Fig. 2E), and the results showed that R^2^ and Q^2^ on the left were both smaller than the initial R^2^ and Q^2^ on the right, indicating that the model was reliable and could be used for subsequent lipid metabolism analysis [27].

**Fig. 3:**
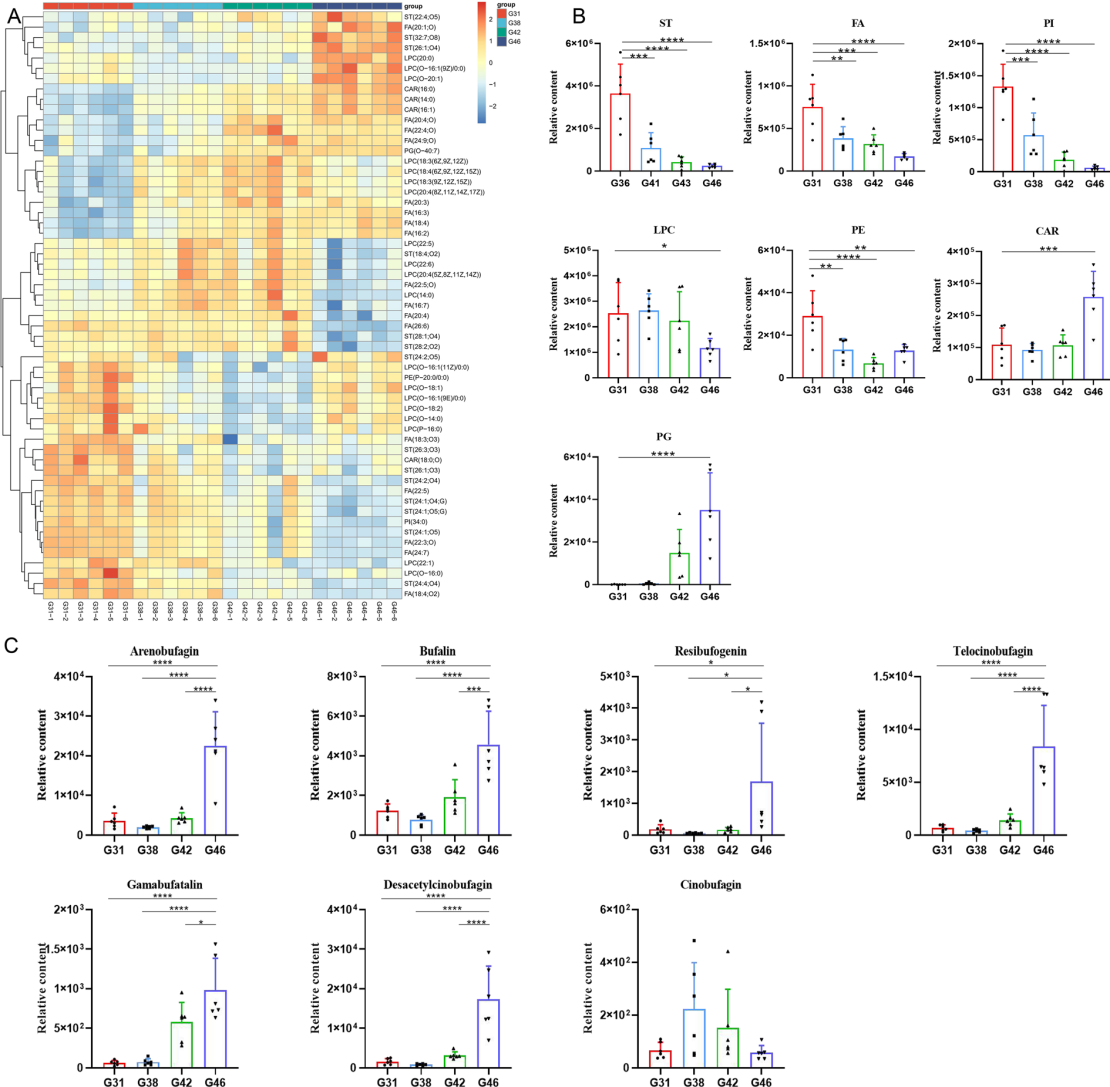
Analysis of lipidome differences during four stages of *B. gargarizans*. **A** Heat map of differential lipid metabolism. **B** Comparison of relative amounts in the lipid subgroups. **C** Comparison of the relative quantities of 7 BDs in each group. ****p < 0.0001, ***p < 0.001, **p < 0.001, *p < 0.01

Comparative research of differential lipids in crucial growth stages was performed to elucidate the regulation mechanism of lipid homeostasis for the development of tadpoles. Progenesis QI selected and identified a total of 89 significantly differential lipid molecules (| log_2_FC |> 1 and adjusted P-value < 0.05) from the comparison between G31 vs. G38, G38 vs. G42, and G42 vs. G46 (Additional file 2: Table S2). There were 48 differential lipids between the G31 group and G38 group: 16 for fatty acyls (including FA and CAR), 17 for LPC, 13 for ST, and 1 for PE and PI; 27 differential lipids were discovered in the G38 and G42 group: 12 for fatty acyls (including FA and CAR), 8 for ST, 5 for LPC, and 1 for PI and PG; 61 lipids were found to be significantly different between the G42 and G46 groups: 14 for fatty acyls (including FA and CAR), 26 for LPC, 18 for ST, and 1 for PE, PG, and PI. To show the variation of differential lipids in tadpoles over time, a heat map was drawn to visualize the tissue distribution (Fig. 3A). Significant changes in the relative quantities of differential lipids at different growth stages suggested the significance and representativeness of the identified lipid metabolites. The clustering of repeats presented satisfactory biological reproducibility; therefore, differential lipids were classified and analyzed by accumulation (Fig. 3B and Additional file 8: Fig. S1). The most significant proportion of ST was measured at the G31 stage, steadily decreased as development proceeded, and included a large number of bile acids, such as apocholic acid (ST 24:2;O4), 7-ketodeoxycholic acid (ST 24:2;O5), glycochenodeoxycholic acid (ST 24:1;O4;G), and ursocholic acid (ST 24:1;O5). The same trend in ST variation was observed for FA and PI, while PG gradually accumulated from the G31 to G46 stage. PE gradually decreased from G31 to G42, but the expression began to callback at the G46 stage, and unlike the others, there was a significant increase or decrease in the relative amounts of CAR and LPC at the G46 stage.

### Accumulation patterns of major BDs in *B. gargarizans* during metamorphosis

By matching retention times and mass-to-charge ratios of the standards, the relative quantities of the major BDs at four key stages were determined from the mass spectrometry data, including cinobufagin, bufalin, resibufogenin, arenobufagin, desacetylcinobufagin, telocinobufagin, and gambufatalin (Fig. 3C). There was a clear accumulation trend, where BDs significantly increased during the entire metamorphosis process except for cinobufagin. Their relative difference in amounts was evident at different stages. In Fig. 3C, arenobufagin was the highest at the G46 stage, followed by desacetylcinobufagin and telocinobufagin, while the lowest amount was for cinobufagin. The shallow level of cinobufagin did not affect the accumulation trend of the total BDs. The corresponding information is summarized in Additional file 3: Table S3.

### Gene regulation process of body remodeling in *B. gargarizans*

To enhance our understanding of the molecular mechanisms involved in the body remodeling regulation of *B. gargarizans*, transcriptome sequencing was performed using the Illumina NovaSeq 6000 platform. After raw data filtering and examination of the sequencing error rate, 78.20 GB of clean data were obtained, with 6.03–7.18 Gb of clean data and a Q30 score of at 93.62–94.45% for each sample. The GC content was distributed in the range of 46.42–47.49% (Additional file 4: Table S4). A boxplot showed trends in transcript level changes in the 12 samples that indicated that the data distribution of gene expression levels in the samples was the same basically (Additional file 9: Fig. S2A). After quality control, the cleaned sequences were aligned to the *B. gargarizans* (Asiatic toad) genome (NCBI_ASM1485885v1, reference genome), with a matching rate of 73.24–77.53%, indicating that the transcriptome data could be further analyzed [1].

A correlation heat map (Additional file 9: Fig. S2B) and the comprehensive PCA (Additional file 9: Fig. S2C) demonstrated satisfactory biological repeatability and significant differences between groups. Moreover, genes were functionally annotated in the GO (Gene Ontology) and KEGG databases. Based on the GO database, 34,129 genes were annotated, and based on the KEGG database, 16.202 genes were functionally annotated. Pairwise comparisons identified four stages of differentially expressed genes (DEGs). As shown in Additional file 9: Fig. S2D and E, 6731 (3626 up and 3105 down), 16,497 (7802 up and 8695 down), and 18,821 (9968 up and 8853 down) DEGs were identified by transcriptome sequencing analysis between different growth groups and the control G31 group. Of these DEGs, 1444 genes were consistently upregulated, and 1385 genes were consistently downregulated, indicating that *B. gargarizans* activated the expression of numerous genes to promote self-development and metamorphosis [28].

The DEGs of G31 vs. G38, G38 vs. G42, and G42 vs. G46 were focused on, including 6731 DEGs (3626 upregulated and 3105 downregulated) of G31 vs. G38, 8627 DEGs (3616 upregulated and 5011 downregulated) of G38 vs. G42, and 9,150 DEGs (5192 upregulated and 3958 downregulated) of G42 vs. G46. The GO enrichment analysis was carried out to elucidate the specific biological functions of the DEGs in the three comparisons (G31 vs. G38, G38 vs. G42, and G42 vs. G46). The top 20 significantly enriched GO terms concerning cellular component (CC), molecular function (MF), and biological process (BP) are shown in Additional file 5: Table S5 and Fig. S3. GO analysis indicated that *B. gargarizans* could effectively regulate peptidase activity and the specific combination of vitamin D, iron ion, and heme to facilitate optimal muscle content, digestion, and metabolism mode, allowing its body to more optimally adapt to metamorphosis processes [29–32]. To confirm the biological pathways of metamorphosis, all DEGs were also assigned to the KEGG database for KEGG pathway analysis (Additional file 6: Table S6 and Fig. S4). The results suggested that the changes in lipid metabolism and energy metabolism provided conditions for *B. gargarizans* as development proceeded.

Moreover, in the present study, the focus was on the detailed functions of the common DEGs (1444 upregulated and 1385 downregulated) from three comparison groups (G31 vs. G38, G31 vs. G42, and G31 vs. G46), as common DEGs were more related to metamorphosis development of *B. gargarizans*. For upregulated DEGs, GO terms were mainly enriched in sarcomere organization, methylation, and muscle contraction in BP; myosin filament, organelle membrane, and troponin complex in CC; and monooxygenase activity in MF (Additional file 12: Fig. S5A). For downregulated DEGs, GO terms were mainly enriched in digestion, cholesterol catabolic process, and peptide catabolic process in BP; brush border membrane and apical plasma membrane in CC; and serine-type endopeptidase activity and iron ion binding in MF (Additional file 12: Fig. S5B).

To further understand the pathways involved in differentially expressed genes, the KEGG database was used to identify DEG-enriched pathways. The results showed that DEGs were significantly enriched in 74 KEGG pathways (36 for upregulated DEGs, 22 for downregulated DEGs, and 16 for up- and downregulated DEGs). These pathways were divided into five levels: metabolism, human diseases, organic systems, environmental information processing, and cellular processes. Of these, nicotinate and nicotinamide metabolism, the estrogen signaling pathway, *Staphylococcus aureus* infection, retinol metabolism, fat digestion, and absorption were the top five enriched pathways in upregulated DEGs. In contrast, protein digestion and absorption, pancreatic secretion, primary bile acid biosynthesis, influenza A, and neuroactive ligand-receptor interaction rank in the top five pathways in downregulated DEGs (Additional file 12: Fig. S5C and D).

### Gene regulation process of lipid changes in *B. gargarizans*

To understand the differences in lipid synthesis during metamorphosis (G31–G46), transcriptomics and lipidomics data were integrated for analysis. Pearson analysis was performed to assess the correlation of 89 differential lipids with DEGs from pairwise comparisons (G31 vs. G38, G31 vs. G42, and G31 vs. G46). Differential lipid-related DEGs were shown in Additional file 7: Table S7. The related DEGs with Pearson's correlation coefficient (PCC) greater than 0.8 were selected for KEGG enrichment analysis (Additional file 13: Fig. S6); they were mainly enriched in “primary bile acid biosynthesis (*P*-value = 4.40E−24),” “PPAR signaling pathway (P-value = 1.10E−23),” “cholesterol metabolism (P-value = 8.20E−17),” “steroid hormone biosynthesis (P-value = 9.49E−17),” “vitamin digestion and absorption (P-value = 9.41E−16)” and other pathways, among which most DEGs were involved in the “PPAR signaling pathway” (ListHit = 37).

**Fig. 4:**
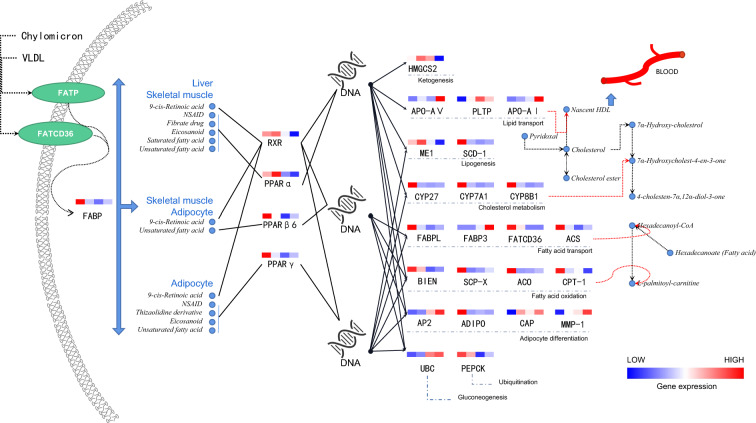
Differential lipid-related DEGs regulate lipid metabolism in the PPAR signaling pathway. The colors of each cell from left to right in the heatmaps represent the gene expression trends among G31, G38, G42, and G46. Gene expression levels are expressed in FPKM values. Fatty acid binding protein (FABP), retinoid x receptor (RXR), peroxisome proliferator-activated receptor (PPAR), 3-hydroxy-3-methylglutaryl-coa synthase 2 (HMGCS2), apolipoprotein (Apo), phospholipid transfer protein (PLTP), malic enzyme 1 (ME1), stearoyl-CoA desaturase (SCD), cytochrome p450 family 27 (CYP27), cytochrome p450 family 7 subfamily a member 1 (CYP7A1), cytochrome p450 family 8 subfamily b member 1 (CYP8B1), acyl-CoA synthetase (ACS), cd36 antigen (FATCD36), enoyl-CoA hydratase (BIEN), sterol carrier protein 2 (SCP-X), acyl-CoA oxidase (ACO), carnitine O-palmitoyltransferase 1 (CPT1), adipocyte (AP2), adiponectin (ADIPO), sorbin and SH3 domain-containing protein 1 (CAP), matrix metalloproteinase-1 (MMP), ubiquitin C (UBC), and phosphoenolpyruvate carboxykinase (PEPCK)

Peroxisome proliferator-activated receptors (PPARs) are nuclear hormone receptors that regulate lipid metabolism, cell differentiation, energy metabolism, and inflammatory response [33]. PPARγ can upregulate the expression of the muscle isoform of carnitine palmitoyltransferase I (CPT1) and FA β-oxidation. It can also regulate the metabolic pathway of cholesterol and polyunsaturated fatty acids by changing the expression of CYP7A1/CYP8B1 [34–36]. As a critical transcription factor in energy metabolism, PPARγ can provide energy for metamorphosis. In the process of G31-G46, the expression of PPARγ, α, and β/δ significantly decreased; the downregulated retinoid X receptor (RXR) also formed heterodimers PPAR-RXR that can combine with the promoter or heterodimers PPAR-RXR through heterodimerization with PPARα, β/δ, or γ to regulate lipid metabolism, fat formation, and maintain metabolic homeostasis [37]. Fig. 4 demonstrates the primary regulatory process of the PPAR signaling pathway during metamorphosis.

The DEGs related to differential lipid and BD accumulation were significantly enriched in the PPAR signaling pathway. To validate the reliability of RNA-Seq data, 6 DEGs with differential lipid correlation coefficients greater than 0.9 (| PCC |≥ 0.9) were selected and examined by qRT-PCR. Except for FABPL in G38 and G42, the expression trends of the selected DEGs were consistent with the Illumina sequencing results (Additional file 14: Fig. S7), indicating the reliability and dependability of the RNA-Seq data.

### Identification of key pathways and genes for BD accumulation/synthesis in *B. gargarizans*

A relatively low level of cinobufagin was not considered based on the assay results of BDs. There were significant differences in the relative quantities of other BDs at the G46 stage compared to other critical stages in the metamorphosis. Therefore, G46 was compared with G31, G38, and G42, and identification was achieved for 3260 common DEGs associated with BD accumulation (Additional file 15: Fig. S8A). GO and KEGG enrichment analyses were performed for these DEGs. The top 30 significantly enriched GO terms concerned CC, MF, and BP (Additional file 15: Fig. S8B). The 3260 DEGs with enriched GO terms were mainly involved in digestion, positive regulation of cell division, negative regulation of endopeptidase activity, polysaccharide digestion, polysaccharide catabolic process, maintenance of gastrointestinal epithelium (BP), extracellular space, extracellular region, myosin filament, myofibril, organelle membrane (CC), serine-type endopeptidase inhibitor activity, serine-type endopeptidase activity, heme binding, aromatase activity, metalloendopeptidase inhibitor activity, iron ion binding, motor activity, aspartic-type endopeptidase activity, and peptidase inhibitor activity (MF). The top 30 significantly enriched KEGG terms are shown in Additional file 15: Fig. S8C, with 3260 DEGs mainly involved in protein digestion and absorption, pancreatic secretion, IL-17 signaling pathway, influenza A, neuroactive ligand-receptor interaction, tight junctions, pathogenic *Escherichia coli* infection, salmonella infection, fat digestion and absorption, and retinol metabolism. The results of these enrichment analyses were too broad, and the range of genes associated with BDs still required narrowing.

**Fig. 5:**
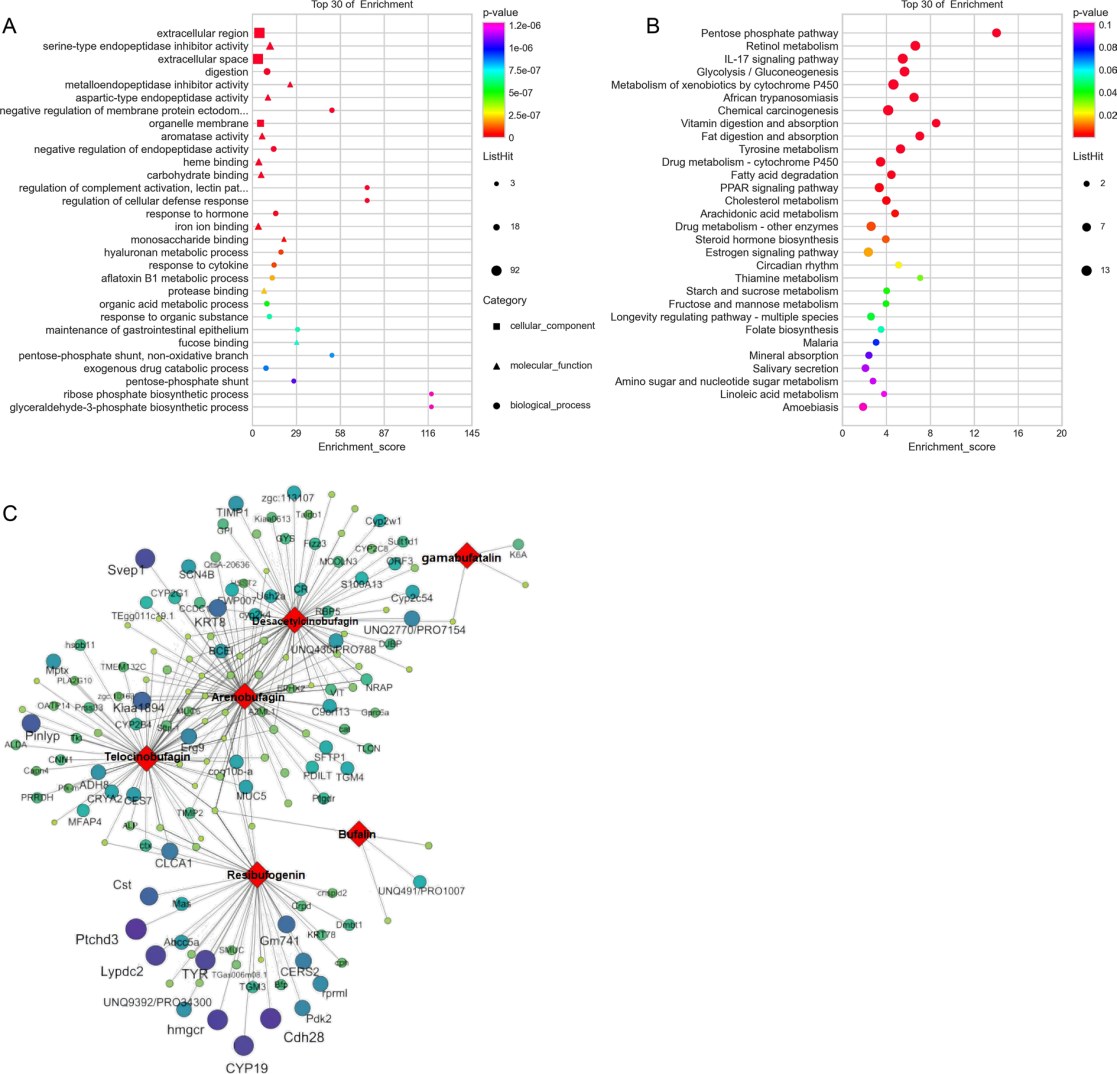
Regulatory analysis of BD accumulation. **A** and **B** The top 30 GO terms and KEGG pathways of significant enrichment for the BD-related DEGs are shown. **C** The top 300 DEGs with the highest correlation coefficients were plotted as co-occurrence networks with bufadienolides. The red diamonds represent BDs, and the circles represent BD-related DEGs. Darker colors and larger circles indicate stronger correlations

To determine the regulatory genes of BD accumulation, Pearson analysis was used to identify DEGs strongly correlated with BDs (|PCC| of ≥ 0.9) to narrow the range. 689 DEGs were identified and subjected to GO and KEGG enrichment analysis. The results showed that DEGs were significantly enriched in pathways such as the “pentose phosphate pathway,” “retinol metabolism,” and “metabolism of xenobiotics by cytochrome p450.” Furthermore, some of the DEGs were enriched in GO terms such as “aromatase activity,” “IL-17 signaling pathway,” “carbohydrate binding,” “iron ion binding,” and “monosaccharide binding” (Fig. 5A and B), which provided a molecular function for further study on the genes related to bufadienolides. “Pentose phosphate pathway” and “aromatase activity” are critical biosynthetic pathways [38, 39], and the DEGs involved in the pathways (GO term) included CYP3A29, CYP2K1, CYP2B4, CYP2D15, CYP2K4, CYP2F3, CYP2C54, CYP2A10, CYP2G1, CYP2H2, PGDH, PFK-M, TKT, ALDA, TALDO1, and GPI.

Pearson analysis was used to evaluate the strength of the correlation between DEGs and BDs, and the top 150 DEGs with the highest correlation coefficients were plotted as co-occurrence networks with BDs (Fig. 5C). In Fig. 5C, the gene correlation coefficient related to resibufagin was higher; desacetylcinobufagin, arenobufagin, and telocinobufagin were more closely related and possessed a more significant number of potential regulatory genes, with bufalin and gamabufatalin having fewer related genes. The accumulation pattern of cinobufagin was different from that of the other BDs. Thus, cinobufagin was not selected for preparing the co-occurrence networks of the common DEGs.

### Visualization of the distribution of BDs in *B. gargarizans*

To understand how BDs are synthesized and accumulated in toads during metamorphosis, DESI-MSI was used to create for the first time a tissue distribution map of BDs in whole-body cryosections of juvenile toads (G46 stage). The workflow of the tissue distribution study of BDs in toads is summarized in Additional file 16: Fig. S9. Two sagittal sections and one coronal section were examined, and light microscopy analysis was used to compare adjacent sections of the sample to determine the BD-enriched organization. Additional file 17: Fig. S10 shows that the organs were well differentiated, indicating that the cryosection method and mass spectrometry imaging conditions for this experiment were suitable. They are feasible for studying the tissue distribution of BDs in this model.

**Fig. 6:**
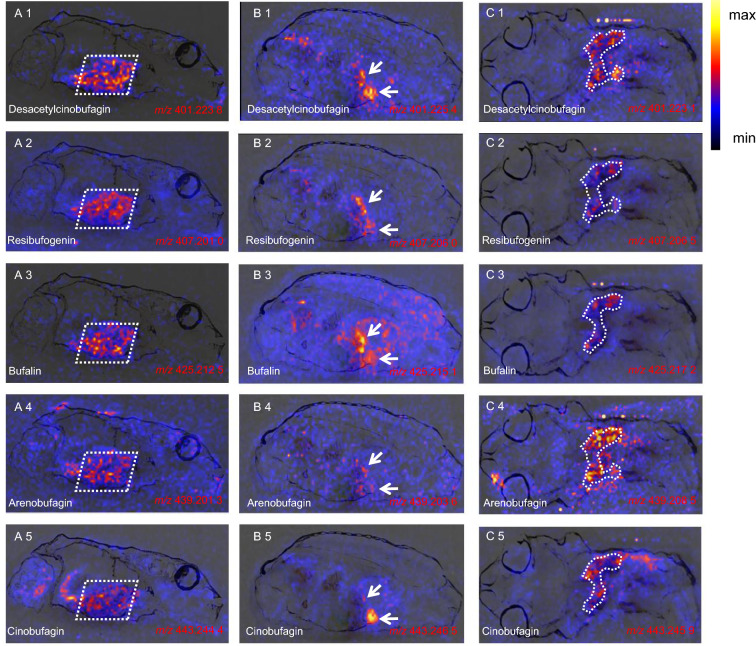
The results of DESI-MSI. The mass spectrometry imaging of desacetylcinobufagin, resibufogenin, bufalin, arenobufagin, and cinobufagin in the three frozen sections in positive ion mode. **A** represents the left sagittal sections (sagittal section **A**), **B** represents the mid-sagittal sections (sagittal section **B**), and **C** represents the horizontal sections (horizontal section **C**). The corresponding information is summarized in Table 1

The results of mass spectrometry imaging are shown in Fig. 6, and include desacetylcinobufagin, resibufogenin, bufalin, arenobufagin, and cinobufagin. Although the response strengths of the five BDs were different, they were all enriched in the same tissues. Fig. 6A(1–5) shows that these BDs were concentrated in the liver of toads but not in the immature parotid gland, suggesting that the juveniles, unlike the adults, do not accumulate BDs in the immature parotid gland but produce a large number of BDs in the liver. Not only did Fig. 6B(1–5) support this result, but it was also found that BDs collected in the gallbladder. Cinobufagin was the most obvious in Fig. 6B5. The function of the gallbladder is to receive bile secreted by hepatocytes. The finding that a large number of BDs were enriched in the liver suggested that BDs were likely to be produced by hepatocytes and accumulate in the gallbladder. A horizontal section was also examined, and BDs were still mainly distributed in liver tissue (Fig. 6C). Optical microscopy analysis using a Zeiss microscope (bright field, top-illumination) to observe the position of each organ in the whole-body sections confirmed that these tissues were derived from the liver (Additional file 17: Fig. S10a–c). Additionally, most of the metabolic pathways involved in BD-related DEGs in Fig. 5B are related to the liver. Thus, the liver plays a crucial role in the biosynthesis and accumulation of BDs.

**Table 1:** Information of 5 mainly BDs identified in DESI-MSI

Name	Proposed formula	Molecular ions	Measured mass	Calculated mass (sagittal section A)	Calculated mass (sagittal section B)	Calculated mass (horizontal section A)
Desacetylcinobufagin	C_24_H_33_O_5_	[M + H]^+^	401.223 8	401.232 3	401.225 4	401.223 1
Resibufogenin	C_24_H_33_O_4_	[M + Na]^+^	407.201 0	407.219 2	407.206 0	407.206 5
Bufalin	C_24_H_35_O_4_	[M + K]^+^	425.212 5	425.208 8	425.215 1	425.217 2
Arenobufagin	C_24_H_33_O_6_	[M + Na]^+^	439.201 3	439.188 1	439.203 6	439.208 5
Cinobufagin	C_26_H_35_O_6_	[M + H]^+^	443.244 4	443.242 8	443.246 5	443.245 9

## Discussion

To adapt to life on land, toads must complete morphological changes and remodeling of organs and cell types in water [40]. In this study, the changes in the lipidome and transcriptome during toad development and metamorphosis, as well as the accumulation characteristics of BDs, were reported. The results showed that the lipid and gene expression in larvae were markedly up- or downregulated in response to body changes during metamorphosis, and BDs continued to increase from G38 and peaked at G46. Additionally, the distribution of BDs in juveniles was observed using whole-body cryosections and DESI-MSI. The results indicated that the liver plays a vital role in the biosynthesis and accumulation of BDs.

### Lipid and transcriptional changes in *B. gargarizans* during metamorphosis

89 differential lipids were identified in this process, and the total relative content of differential lipids gradually decreased (Additional file 8: Fig. S1). The most apparent decrease in differential lipids was in sterol lipids (ST), which mainly include a large quantity of bile acids, such as apocholic acid (ST 24:2;O4), 7-ketodeoxycholic acid (ST 24:2;O5), glycochenodeoxycholic acid (ST 24:1;O4;G), and ursocholic acid (ST 24:1;O5). The enrichment analysis results for DEGs (G31 vs. G38, G38 vs. G42) also suggested a decrease in bile secretion and tyrosine metabolism, indicating that loss of appetite and decreased food intake occured during metamorphosis (Additional file 11: Fig. S4B and D).

Before metamorphosis, tadpoles actively ate to provide adequate nutritional preparation and induce the process of metamorphosis [9, 41]. After metamorphosis began, the hormones leptin and stress neuropeptide corticotropin-releasing factor acted on feeding control centers in the hypothalamus to inhibit feeding [42]. Decreased eating not only assisted with intestinal remodeling, but also reduced the body fat of toads, providing favorable conditions for adapting to terrestrial life. However, large amounts of energy were still required for the morphological changes during metamorphosis. The tail of the tadpole began to be absorbed at the G38 stage, and the relative quantities of fatty acids (FAs) were also decreasing, indicating that the tail changed from an energy-consuming organ to an energy-supplying organ, replacing the fat depot as an energy source for metamorphosis [9]. However, the low appetite phase was likely to last longer than the tail absorption period. Amino acids or proteins that flowed from the tail to the liver were adequately mobilized as extrahepatic nutrients due to the inconvenience of bulk storage. KEGG enrichment analysis showed that the gluconeogenesis function of tadpoles in the metamorphosis climax stage was continuously enhanced (Additional file 11: Fig. S4C and E). Enhanced gluconeogenesis provided energy for the low appetite phase following complete absorption of the tail, and the increased CAR and decreased FA have suggested that starvation-induced enhanced lipid catabolism in the liver and muscle [43].

Clinically, PG and PI are auxiliary indicators for the diagnosis of fetal lung maturity. In Fig. 3B, the opposite trend was observed for them. The ratio of PG to PI in the amniotic fluid increases gradually as the fetal lung develops to maturity. In this study, to facilitate respiration in air, the primary larval gills had transformed into lung breathing through morphological innovations, which was also a process of lung maturation. The relative quantities of lyso-phosphatidyl cholines (LPC) decreased at G46. The main component, LPC 20:4(5Z,8Z,11Z,14Z), is derived from arachidonic acid, and its level is linearly related to the number of preserved beta cells [44]. The consumption of LPC 20:4(5Z,8Z,11Z,14Z) from G42 to G46 may be caused by the remodeling of the endocrine pancreas, while the results of the KEGG enrichment analysis showed that pancreatic secretion was reduced since the G31 stage, indicating that beta cell death may be simply the final process of pancreatic remodeling. In the adult *Xenopus* pancreas, islets contain only insulin-producing beta cells, and it has been proposed that the maturation of the pancreatic system occurs at the metamorphosis climax [41]. However, beta cells do not replicate and are involved in limited cell death and changes in insulin expression during this process. LPC22:6 confers protective effects on the heart and brain, and supplementing it in cardiac arrest rats can aid in cardiac resuscitation. Thus, the high consumption after G31 may promote cardiac development. The KEGG enrichment analysis also showed that cardiac muscle contraction was continuously enhanced during metamorphosis (Additional file 12: Fig. S5B) [45].

Pinelli et al. performed a comparative analysis of apoptotic and mitotic indices in the developing eye of toad larvae, and they found that the peaks of apoptosis and proliferation in retinal cells appeared in G31 and G38 [46], which was consistent with our enrichment analysis results (Additional file 11: Fig. S4A and C). PE(P-20:0/0:0), which is a marker of apoptotic cells [47, 48], was highly expressed when the organ began to remodel, gradually decreased with redevelopment, and finally increased again when the tail was apoptotic (Fig. 3B).

### Mechanism of regulating differential lipids and BDs

The metabolism and composition of lipids were comprehensively analyzed in 4 different developmental stages for tadpoles, and it was found that the genes relevant to differential lipids were mainly enriched in the PPAR signaling pathway. There are three different isoforms of peroxisome proliferator-activated receptors (PPARs) (i.e., PPARα, PPARβ/δ, and PPARγ), and they function as ligand-activated transcription factors with DNA-binding domains that recognize response elements in the promoter region of specific target genes linked to inflammation, apoptosis, cell proliferation, and differentiation [49]. The PPAR signaling pathway was important in regulating lipid metabolism during metamorphosis.

In the present study, the sharp decrease in the expression of genes related to fatty acid oxidation after G31 was due to the priority consumption of amino acids and glycogen rather than liver lipids. The expression trend of cholesterol metabolism-related genes supported this point. Starvation will lead to ketone production, and the increase in ketone bodies induced proliferation, differentiation, and apoptosis in intestinal crypts, which accelerated the remodeling process.

Extrahepatic tissues also used ketone bodies to generate large amounts of acetyl CoA, which can inhibit pyruvate dehydrogenase activity, limit glucose consumption, activate pyruvate carboxylase, and promote gluconeogenesis. This can reduce glucose uptake by extrahepatic tissues to ensure that sufficient glucose reaches the brain tissue and red blood cells. In addition, brain tissues cannot use long-chain fatty acids, but ketone bodies can be used for energy [50]. The expression trend of related genes of ketogenesis (HMGCS2) and gluconeogenesis (UBC) also reflected that. The continuous decrease in SCD-1, an essential gene of adipogenesis, inhibited adipogenesis so that the limbs could better control the body after landing.

The PPAR signaling pathway identified by combined lipidomic and transcriptomic analysis reveals the regulatory process of lipids and genes during metamorphosis, which is an excellent method for analyzing the biosynthetic mechanism of BDs. According to the accumulation trend of BDs at the four stages, 3260 DEGs were identified, and 689 DEGs strongly associated with BDs were identified by Pearson correlation analysis. KEGG and GO enrichment analyses were subsequently performed on the strongly related DEGs. The enrichment results prompted two important synthetic pathways for us. Two important synthetic pathways, “pentose phosphate pathway” and “aromatase activity,” were led according to the enrichment results. The primary physiological function of the pentose phosphate pathway (PPP) is the production of NADPH, which provides reducing power for biosynthesis. It is likely that the NADPH produced by PPP assists in converting the parent nucleus of cholesterol into the cis-fused ring of BDs. In addition, “aromatase activity” was related to catalyzing the reduction of aliphatic rings to generate aromatic rings. The DEGs in “aromatase activity” may be critical regulatory genes for the cyclization of cholesterol side chains into rings.

The pathways involved 16 DEGs, of which 10 were derived from cytochrome P450s, including CYP3A29, CYP2K1, CYP2B4, CYP2D15, CYP2K4, CYP2F3, CYP2C54, CYP2A10, CYP2G1, CYP2H2. Cytochrome P450s are monooxygenases that catalyze reactions involving drug metabolism and synthesis of lipids (cholesterol, steroids, etc.). The regulations of cytochrome P450s were required for both the synthesis and accumulation of BDs and the self-protection caused by endogenous BDs. The cytochrome P450s in DEGs were almost all from the CYP2 family. The annotation information and literature indicate that these CYP2s have the following functions in synthesising BDs. Firstly, CYP2F3 has a clear role in dehydrogenation [51]. It may be involved in the dehydrogenation of 3-OH, which is a key step in the isomerization of the steroidal nucleus of cholesterol to cis-fused rings. Secondly, the conversion of cholesterol to BDs also involves hydroxylation at the C-1β, 3β, 5β, 11α, 12α/β, 14β, and 16β sites. CYP2K1, CYP2D15, CYP2A10 and CYP2H2 may be related to that, which can hydroxylate steroids or lipids [52–55]. Lastly, CYP2B4, CYP2C54, CYP2G1, and CYP2H2 were involved in the cyclooxygenase P450 pathway [56], which may play a key role in the generation of unsaturated six-membered lactone ring from bufadienolide. It can be inferred that CYP2s are very important in the biosynthesis of BDs.

### Synthetic sites and distribution of BDs

DESI-MSI combined with whole-body cryosectioning was an effective method to study the tissue distribution and synthesis sites of BDs. This is the first time this method has been applied to studying BD tissue distribution and biosynthesis. The whole-body mapping of BDs in toads by DESI-MSI was the initial step to gaining insight into BD biosynthesis. In Fig. 6A, BDs are characteristically distributed in the liver but not enriched in the immature parotid gland. BDs were most abundant in the parotid glands of adult toads. By stimulating these glands, they can secrete large quantities of Chansu (the crude extract of BDs), which can be used as the raw material for clinical preparations.

Excessive storage of BDs, which may result in excessive peak height in the gland and hinder the observation of other organs and tissues, will not occur when juvenile toad larvae are used as the samples for tissue distribution research. The results in Fig. 6A suggest that BDs can be synthesized in the liver, and the parotid gland may simply be a storage organ for BDs. Garraffo et al. demonstrated that the glands were unable to produce the synthetic precursor cholesterol of BDs when sodium (1-^14^C) acetate and (5-^3^H) mevalonate were co-incubated with toad liver tissue and postauricular gland tissue, respectively [57], which also supported our opinion.

Fig. 6B shows that BDs also had a distinct characteristic distribution in the gallbladder, and the amount of cinobufagin in the gallbladder was much higher than that in the liver. This suggests that the gallbladder may be the first collection organ of BDs, and it is responsible for transporting BDs produced by the liver to the skin and glands, while the BDs of juvenile toads mainly accumulated in the liver and gallbladder. The analysis of horizontal sections also showed that a large number of BDs were enriched in the liver (Fig. 6C), again confirming the importance of the liver in the synthesis of BDs and implicating it as an independent synthesis site of BDs.

## Conclusion

In summary, lipidomics and transcriptomic methods were used to study the metamorphic development of *bufo gargarizans*, demonstrating the diet, energy metabolism, and growth of the organs of toads. The PPAR signaling pathway was mapped as the main lipids regulatory pathway, demonstrated the processes of G31-G46 regulating ketogenesis, adipogenesis, cholesterol metabolism, and fatty acid oxidation. The accumulation trends of seven BDs were identified during the metamorphosis with standards and suggested the key pathways of BDs biosynthesis combined with the transcriptomic data. Furthermore, the study of BDs tissue distribution by DESI-MSI indicated that the liver plays a crucial role in BDs biosynthesis and may be the synthetic site of BDs. Overall, this study provides a valuable data resource for the resource development of *B. gargarizans* and BDs.

## Supplementary Information

**Additional file 1: Table S1.** Specific primer pairs for selected genes.

**Additional file 2: Table S2.** Differential lipids in four stages.

**Additional file 3: Table S3.** BDs semi-quantitative analysis reports.

**Additional file 4: Table S4.** After raw data filtering and examination of the sequencing error rate and GC content distribution.

**Additional file 5: Table S5.** GO enrichment analysis (Top 20).

**Additional file 6: Table S6.** KEGG enrichment analysis (Top 20).

**Additional file 7: Table S7.** Differential lipids related DEGs (TOP 500).

**Additional file 8: Fig. S1.** Cumulative lipids composition.

**Additional file 9: Fig. S2.** Multivariate analysis of the transcriptome in B. gargarizans at different growth stages. (A) The degree of dispersion of the FPKM distribution. The horizontal axis is the sample name, and the vertical axis is for log10(FPKM+1). Box plot elements denote maximum, third quartile, median, first quartile, and minimum. (B) Heatmap for Pearson correlation coefficients (PCC) between samples. The horizontal axis represents the sample name, the vertical axis represents the corresponding sample name, and the color denotes the size of the correlation coefficient. (C) Principal component analysis (PCA) plot of the transcriptomic data from the 12 samples. (D and E) Up- and downregulated differential Venn diagram. Each circle represents a comparison group, the numbers outside the overlaps represent the number of specific DEGs of the comparison group, and the numbers in the circle overlaps represent the number of DEGs common to the comparison group.

**Additional file 10: Fig. S3.** GO enrichment bubble diagram (G31 vs. G38, G38 vs. G42, G42 vs. G46). (A) and (B) showed the top 30 pathways of significant enrichment of the upregulated and downregulated DEGs on GO for G31 vs. G38, (C) and (D) showed for G38 vs. G42, and (E) and (F) showed for G42 vs. G46.

**Additional file 11: Fig. S4.** KEGG enrichment bubble diagram (G31 vs. G38, G38 vs. G32, G42 vs. G46). (A) and (B) showed the top 30 pathways of significant enrichment of the up-and down- regulated DEGs on KEGG for G31 vs. G38, (C) and (D) showed for G38 vs. G42, and (E) and (F) showed for G42 vs. G46.

**Additional file 12: Fig. S5.** Enrichment analysis of DEGs for B. gargarizans metamorphosis. The top 30 GO terms and pathways of significant enrichment for the (A and B) upregulated DEGs and (C and D) downregulated DEGs. The y-axis corresponds to the GO term or KEGG pathway, the x-axis corresponds to the EnrichmentScore, and the size of the point corresponds to the number of differential genes in the term or pathway.

**Additional file 13: Fig. S6.** Enrichment analysis of differential lipid-related DEGs. (A) showed the top 30 GO terms of significant enrichment of the differential lipid-related DEGs on GO;(B) showed the top 30 pathways of significant enrichment of the differential lipid-related DEGs on KEGG.

**Additional file 14: Fig. S7.** Results of RT-qPCR.

**Additional file 15: Fig. S8.** Enrichment analysis of DEGs for BD accumulation. (A) By comparing G46 with G31, G38 and G42 respectively, 3260 common DEGs were identified. (B) and (C) showed the top 30 GO terms and pathways of significant enrichment of the BDs-related DEGs.

**Additional file 16: Fig. S9.** General overview of the major steps in DESI-MSI in the entire toad at the G46 stage. Step 1. After removing the limbs of the toads (G46 stage), whole-body cryosections were created along the sagittal and horizontal planes. Step 2. Serial tissue sections were collected while performing the optical microscopy and DESI-MSI techniques, and the appropriate sections were then selected for testing. DESI-MSI was performed, including generation of mass spectra and conversion to image, and identification of organs and tissues with optical microscopy.

**Additional file 17: Fig. S10.** Identification of organs in DESI-MSI sections. (A–C) Frozen sections selected for DESI-MSI; (D–F) DESI-MSI at a specific mass-to-charge ratio that presents a clear org an position. (a–c) Microscope images of the corresponding parts of A to C. A, D and a representative of the left sagittal sections (sagittal section A); B, E, and b represent the mid-sagittal sections (sagittal section B); C, F, and c represent the horizontal sections (horizontal section C).

## Data Availability

The RNA-seq reads of the toads at the four stages can be obtained from BioProject PRJNA853591. The assembled *Bufo gargarizans* genome ASM1485885v1 was deposited in NCBI under BioProject PRJNA628553.

## Abbreviations

DESI: Desorption electrospray ionization
LC–MS: Liquid chromatography-tandem mass spectrometry
DEGs: Differentially expressed genes
BDs: Bufadienolides
MSI: Mass spectrometry imaging
PPAR: Peroxisome proliferators-activated receptor
G: Gosner stage
KEGG: Kyoto Encyclopedia of Genes and Genomes
GO: Gene ontology
FA: Fatty acids
ST: Sterol
PI: Phosphatidylinositol
LPC: Lyso-posphatidyl choline
PE: Phosphatidylethanolamine
CAR: Carnitines
PG: Phosphatidylglycerol
PCA: Orthogonal partial least-squares discrimination analysis
OPLS-DA: Orthogonal partial least-squares discrimination analysis
RT-qPCR: Quantitative real-time PCR
GC: Guanine and cytosine
PPP: Pentose phosphate pathway
