# Supplementary figures and images for: Lipidomics combined with transcriptomic and mass spectrometry imaging analysis of the Asiatic toad (Bufo gargarizans) during metamorphosis and bufadienolide accumulation
Source: Chin Med. 2022 Nov 4;17:123. doi: 10.1186/s13020-022-00676-7 (PMC9636624; doi:10.1186/s13020-022-00676-7)

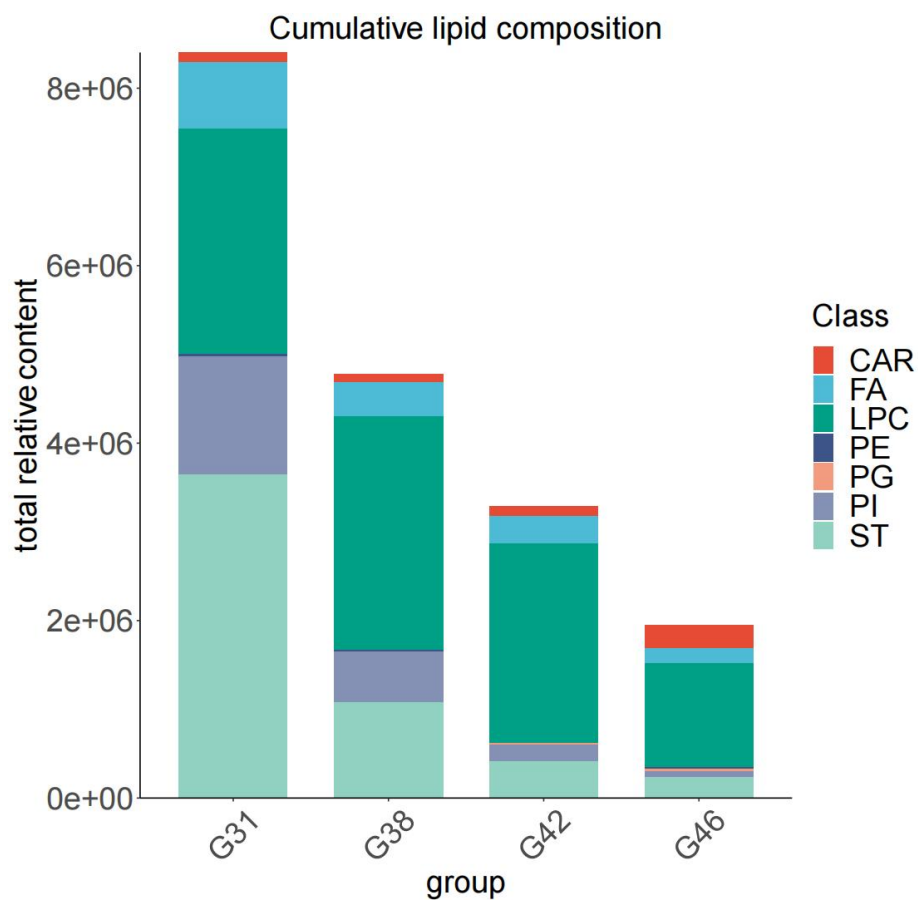

**Fig. S1.** Cumulative lipids composition

Supplement: Supplementary file 8 — Additional file 8: Fig. S1. Cumulative lipids composition. [file 13020_2022_676_MOESM8_ESM.pdf]

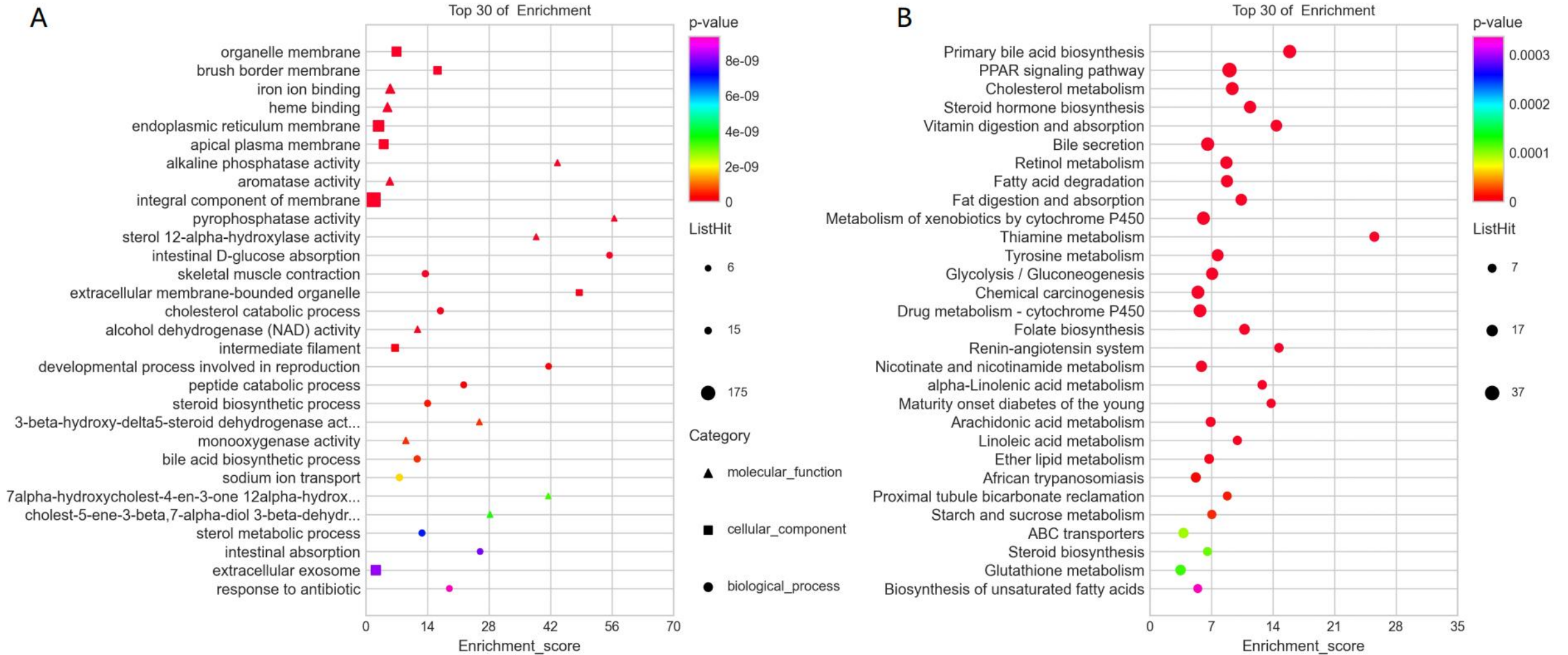

Supplement: Supplementary file 13 — Additional file 13: Fig. S6. Enrichment analysis of differential lipid-related DEGs. (A) showed the top 30 GO terms of significant enrichment of the differential lipid-related DEGs on GO;(B) showed the top 30 pathways of significant enrichment of the differential lipid-related DEGs on KEGG. [file 13020_2022_676_MOESM13_ESM.pdf]

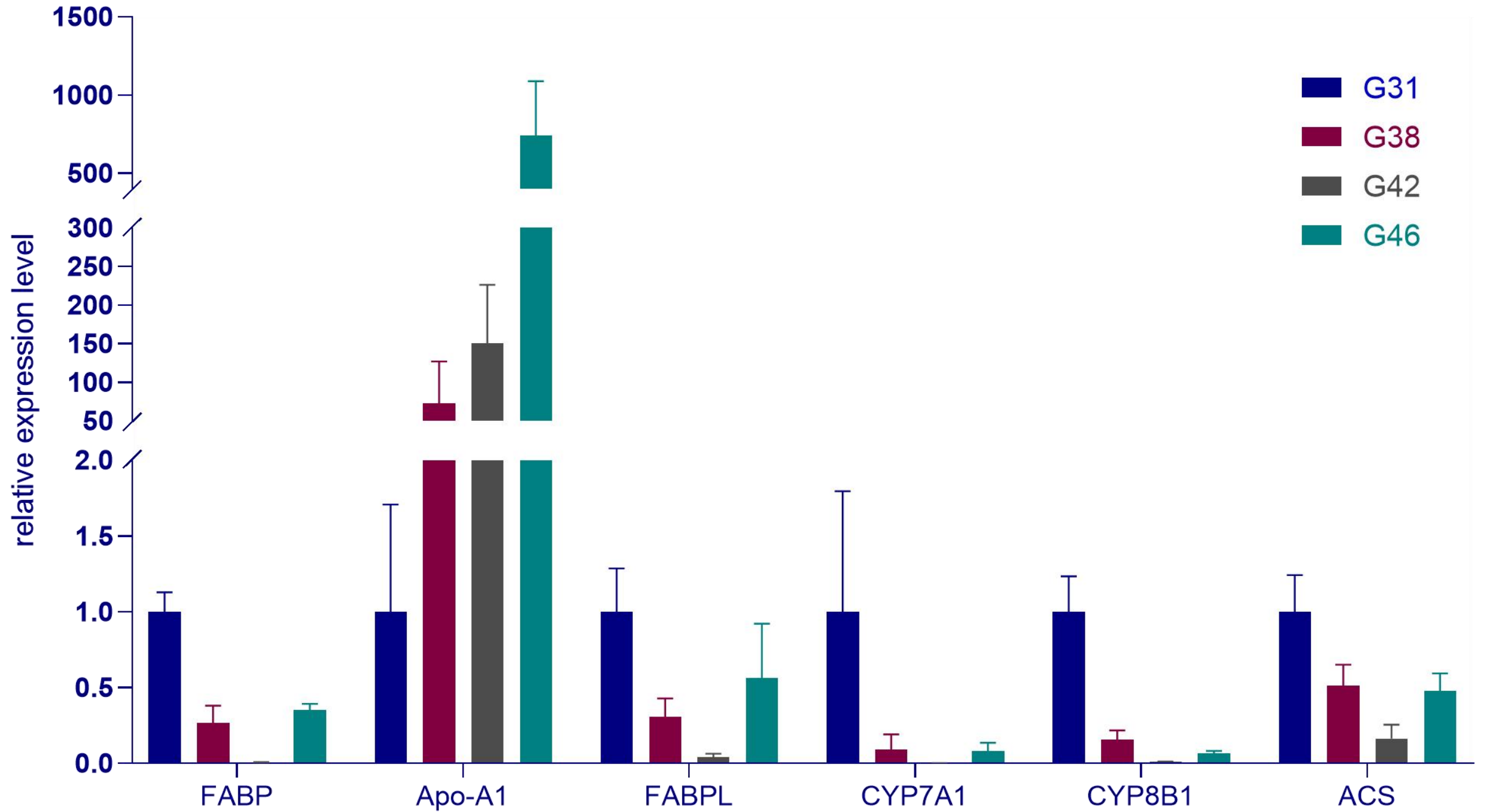

Fig. S7. Results of RT-qPCR

Supplement: Supplementary file 14 — Additional file 14: Fig. S7. Results of RT-qPCR. [file 13020_2022_676_MOESM14_ESM.pdf]
