# Supplementary material for: Lipidomics combined with transcriptomic and mass spectrometry imaging analysis of the Asiatic toad (Bufo gargarizans) during metamorphosis and bufadienolide accumulation
Source: Chin Med. 2022 Nov 4;17:123. doi: 10.1186/s13020-022-00676-7 (PMC9636624; doi:10.1186/s13020-022-00676-7)
Supplement: Supplementary file 9 — Additional file 9: Fig. S2. Multivariate analysis of the transcriptome in B. gargarizans at different growth stages. (A) The degree of dispersion of the FPKM distribution. The horizontal axis is the sample name, and the vertical axis is for log10(FPKM+1). Box plot elements denote maximum, third quartile, median, first quartile, and minimum. (B) Heatmap for Pearson correlation coefficients (PCC) between samples. The horizontal axis represents the sample name, the vertical axis represents the corresponding sample name, and the color denotes the size of the correlation coefficient. (C) Principal component analysis (PCA) plot of the transcriptomic data from the 12 samples. (D and E) Up- and downregulated differential Venn diagram. Each circle represents a comparison group, the numbers outside the overlaps represent the number of specific DEGs of the comparison group, and the numbers in the circle overlaps represent the number of DEGs common to the comparison group. [file 13020_2022_676_MOESM9_ESM.pdf]

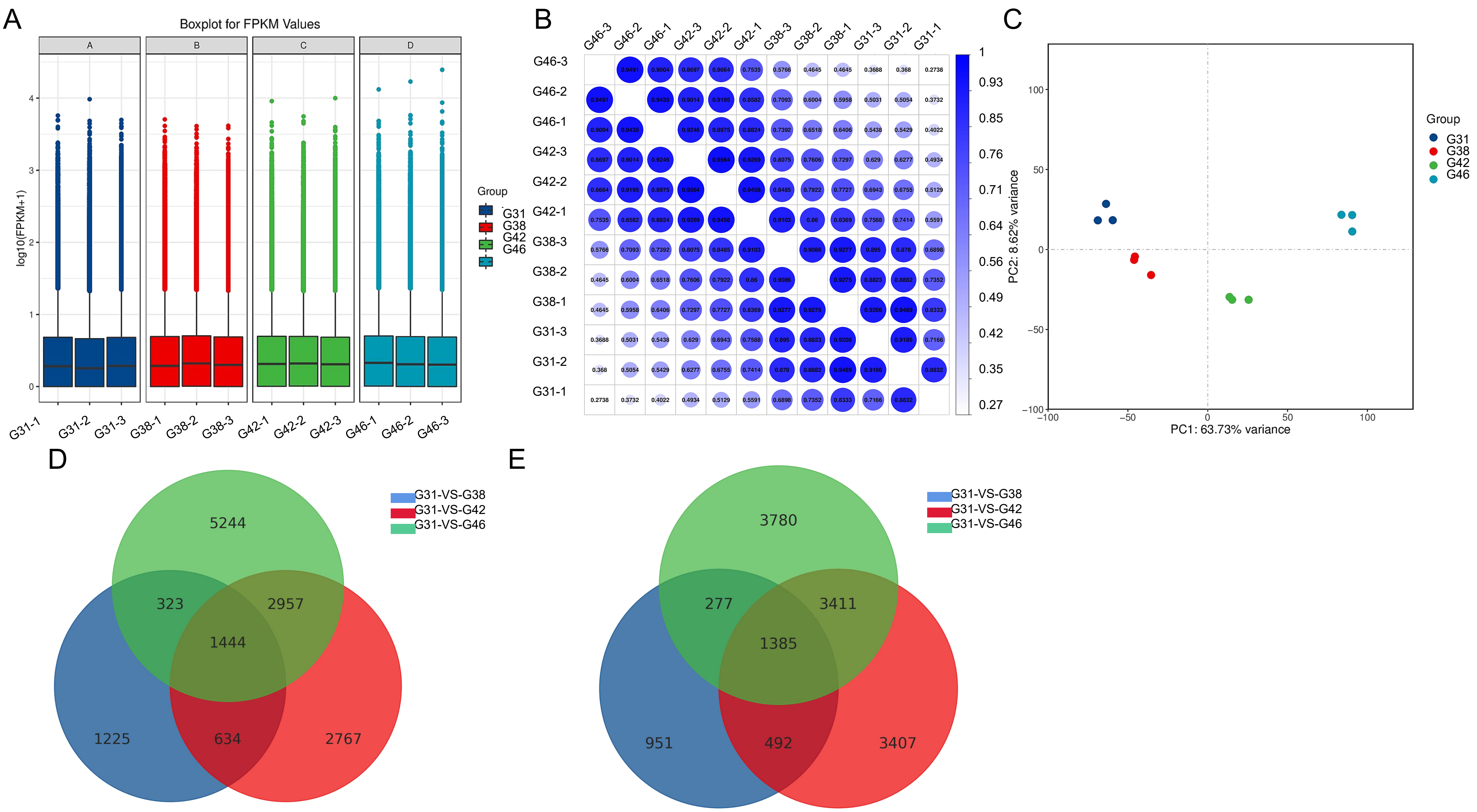

Fig. S2. Multivariate analysis of the transcriptome in *B. gargarizans* at different growth stages. (A) The degree of dispersion of the FPKM distribution. The horizontal axis is the sample name, and the vertical axis is for log10(FPKM+1). Box plot elements denote maximum, third quartile, median, first quartile, and minimum. (B) Heatmap for Pearson correlation coefficients (PCC) between samples. The horizontal axis represents the sample name, the vertical axis represents the corresponding sample name, and the color denotes the size of the correlation coefficient. (C) Principal component analysis (PCA) plot of the transcriptomic data from the 12 samples. (D and E) Up- and downregulated differential Venn diagram. Each circle represents a comparison group, the numbers outside the overlaps represent the number of specific DEGs of the comparison group, and the numbers in the circle overlaps represent the number of DEGs common to the comparison group.
