# Supplementary material for: Lipidomics combined with transcriptomic and mass spectrometry imaging analysis of the Asiatic toad (Bufo gargarizans) during metamorphosis and bufadienolide accumulation
Source: Chin Med. 2022 Nov 4;17:123. doi: 10.1186/s13020-022-00676-7 (PMC9636624; doi:10.1186/s13020-022-00676-7)
Supplement: Supplementary file 10 — Additional file 10: Fig. S3. GO enrichment bubble diagram (G31 vs. G38, G38 vs. G42, G42 vs. G46). (A) and (B) showed the top 30 pathways of significant enrichment of the upregulated and downregulated DEGs on GO for G31 vs. G38, (C) and (D) showed for G38 vs. G42, and (E) and (F) showed for G42 vs. G46. [file 13020_2022_676_MOESM10_ESM.pdf]

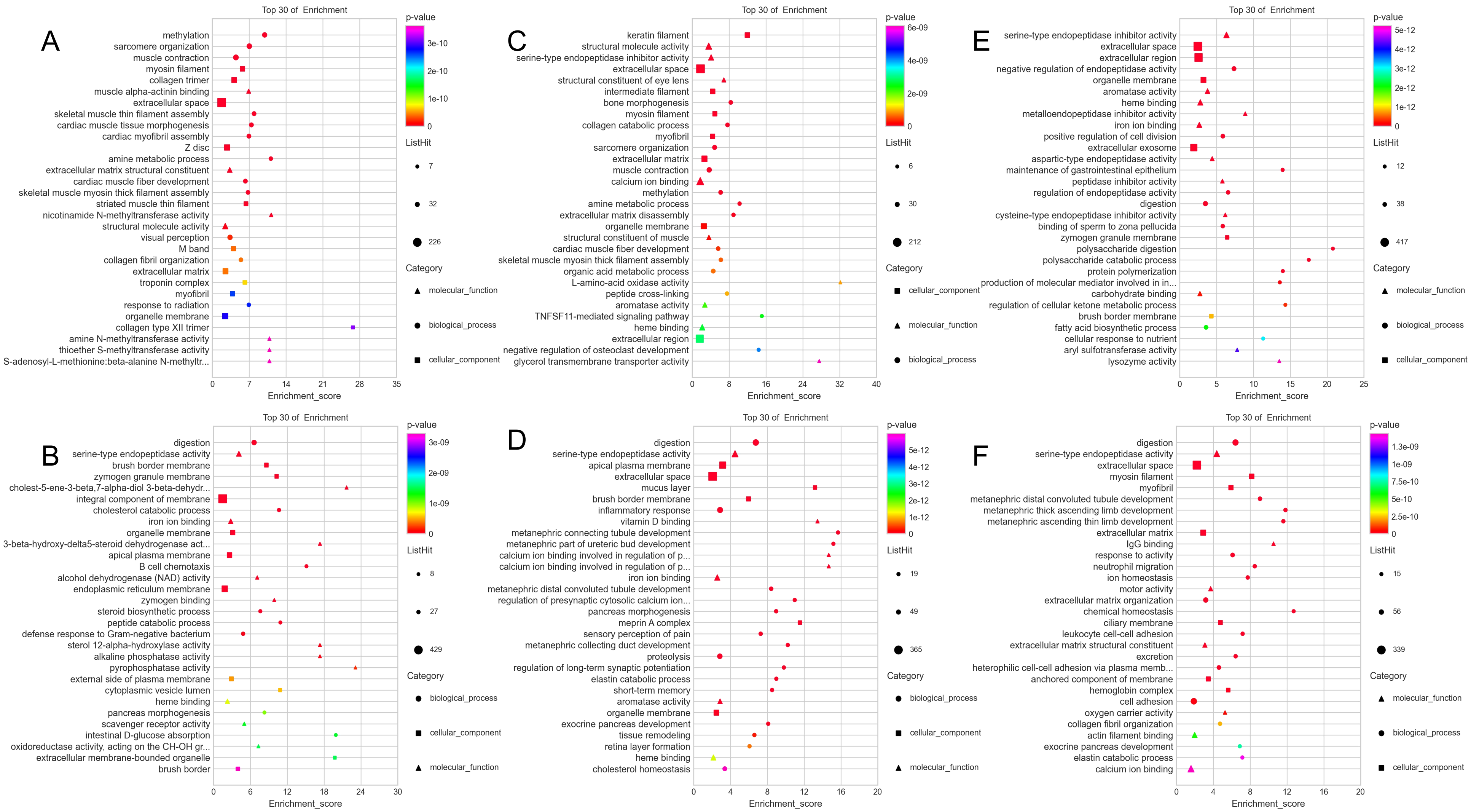

Fig. S3. GO enrichment bubble diagram (G31 vs. G38, G38 vs. G42, G42 vs. G46). (A) and (B) showed the top 30 pathways of significant enrichment of the upregulated and downregulated DEGs on GO for G31 vs. G38, (C) and (D) showed for G38 vs. G42, and (E) and (F) showed for G42 vs.G46.
