# Supplementary material for: Lipidomics combined with transcriptomic and mass spectrometry imaging analysis of the Asiatic toad (Bufo gargarizans) during metamorphosis and bufadienolide accumulation
Source: Chin Med. 2022 Nov 4;17:123. doi: 10.1186/s13020-022-00676-7 (PMC9636624; doi:10.1186/s13020-022-00676-7)
Supplement: Supplementary file 11 — Additional file 11: Fig. S4. KEGG enrichment bubble diagram (G31 vs. G38, G38 vs. G32, G42 vs. G46). (A) and (B) showed the top 30 pathways of significant enrichment of the up-and down- regulated DEGs on KEGG for G31 vs. G38, (C) and (D) showed for G38 vs. G42, and (E) and (F) showed for G42 vs. G46. [file 13020_2022_676_MOESM11_ESM.pdf]

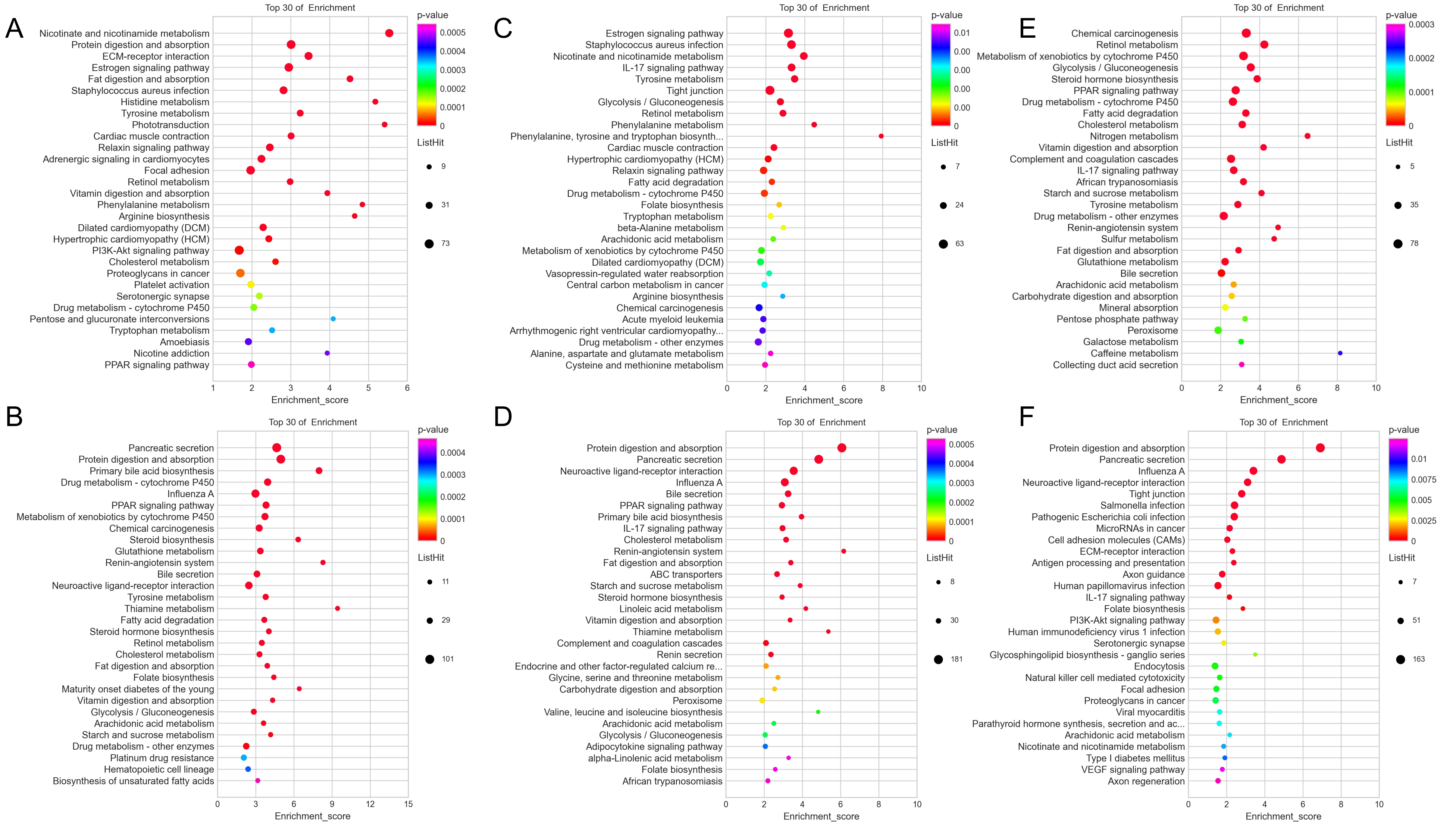

Fig. S4. KEGG enrichment bubble diagram (G31 vs. G38, G38 vs. G32, G42 vs. G46). (A) and (B) showed the top 30 pathways of significant enrichment of the up-and down- regulated DEGs on KEGG for G31 vs. G38, (C) and (D) showed for G38 vs. G42, and (E) and (F) showed for G42 vs. G46
