# Supplementary material for: Lipidomics combined with transcriptomic and mass spectrometry imaging analysis of the Asiatic toad (Bufo gargarizans) during metamorphosis and bufadienolide accumulation
Source: Chin Med. 2022 Nov 4;17:123. doi: 10.1186/s13020-022-00676-7 (PMC9636624; doi:10.1186/s13020-022-00676-7)
Supplement: Supplementary file 12 — Additional file 12: Fig. S5. Enrichment analysis of DEGs for B. gargarizans metamorphosis. The top 30 GO terms and pathways of significant enrichment for the (A and B) upregulated DEGs and (C and D) downregulated DEGs. The y-axis corresponds to the GO term or KEGG pathway, the x-axis corresponds to the EnrichmentScore, and the size of the point corresponds to the number of differential genes in the term or pathway. [file 13020_2022_676_MOESM12_ESM.pdf]

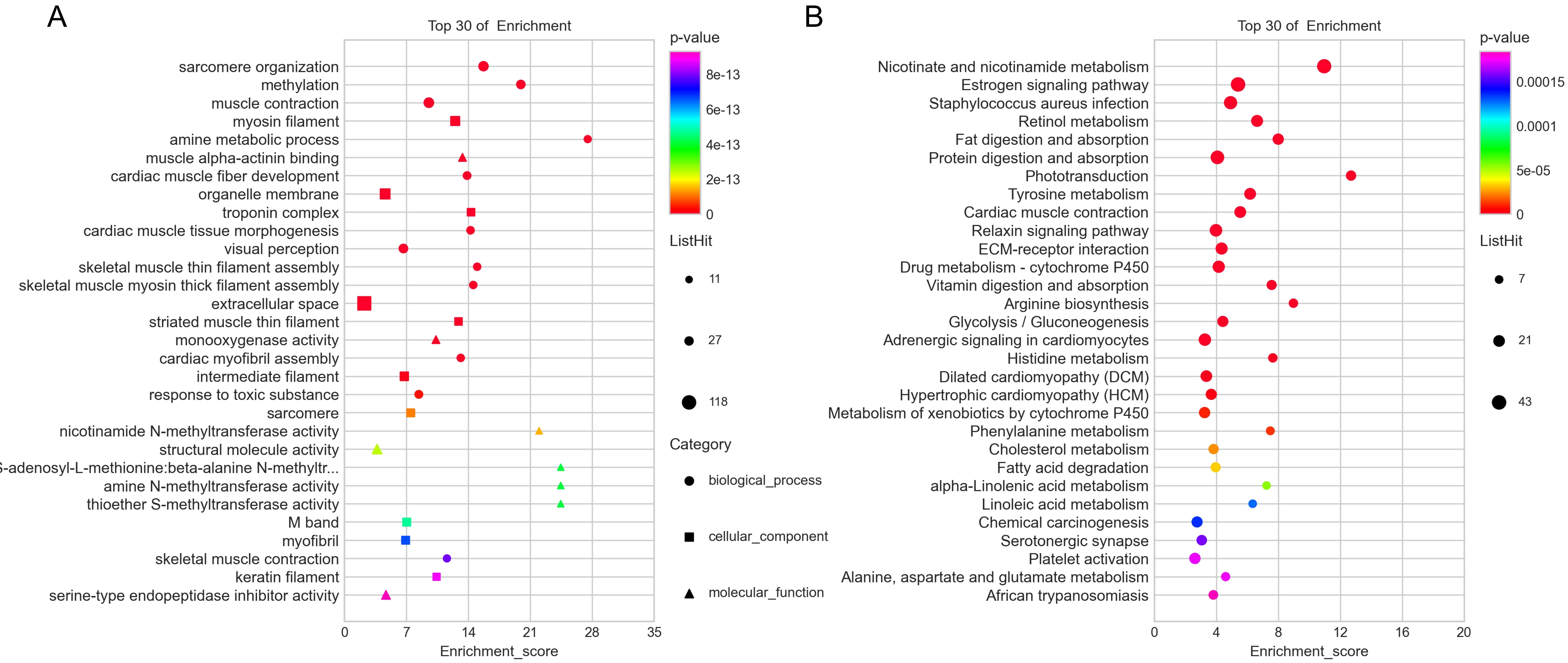
A
Top 30 of Enrichment
sarcomere organization
methylation
muscle contraction
myosin filament
amine metabolic process
muscle alpha-actinin binding
cardiac muscle fiber development
organelle membrane
troponin complex
cardiac muscle tissue morphogenesis
visual perception
skeletal muscle thin filament assembly
skeletal muscle myosin thick filament assembly
extracellular space
striated muscle thin filament
monooxygenase activity
cardiac myofibril assembly
intermediate filament
response to toxic substance
sarcomere
nicotinamide N-methyltransferase activity
structural molecule activity
S-adenosyl-L-methionine:beta-alanine N-methyltr...
amine N-methyltransferase activity
thioether S-methyltransferase activity
M band
myofibril
skeletal muscle contraction
keratin filament
serine-type endopeptidase inhibitor activity
0
7
14
21
28
35
Enrichment_score
p-value
8e-13
6e-13
4e-13
2e-13
0
ListHit
11
27
118
Category
biological_process
cellular_component
molecular_function
B
Top 30 of Enrichment
Nicotinate and nicotinamide metabolism
Estrogen signaling pathway
Staphylococcus aureus infection
Retinol metabolism
Fat digestion and absorption
Protein digestion and absorption
Phototransduction
Tyrosine metabolism
Cardiac muscle contraction
Relaxin signaling pathway
ECM-receptor interaction
Drug metabolism - cytochrome P450
Vitamin digestion and absorption
Arginine biosynthesis
Glycolysis / Gluconeogenesis
Adrenergic signaling in cardiomyocytes
Histidine metabolism
Dilated cardiomyopathy (DCM)
Hypertrophic cardiomyopathy (HCM)
Metabolism of xenobiotics by cytochrome P450
Phenylalanine metabolism
Cholesterol metabolism
Fatty acid degradation
alpha-Linolenic acid metabolism
Linoleic acid metabolism
Chemical carcinogenesis
Serotonergic synapse
Platelet activation
Alanine, aspartate and glutamate metabolism
African trypanosomiasis
0
4
8
12
16
20
Enrichment_score
p-value
0.00015
0.0001
5e-05
0
ListHit
7
21
43

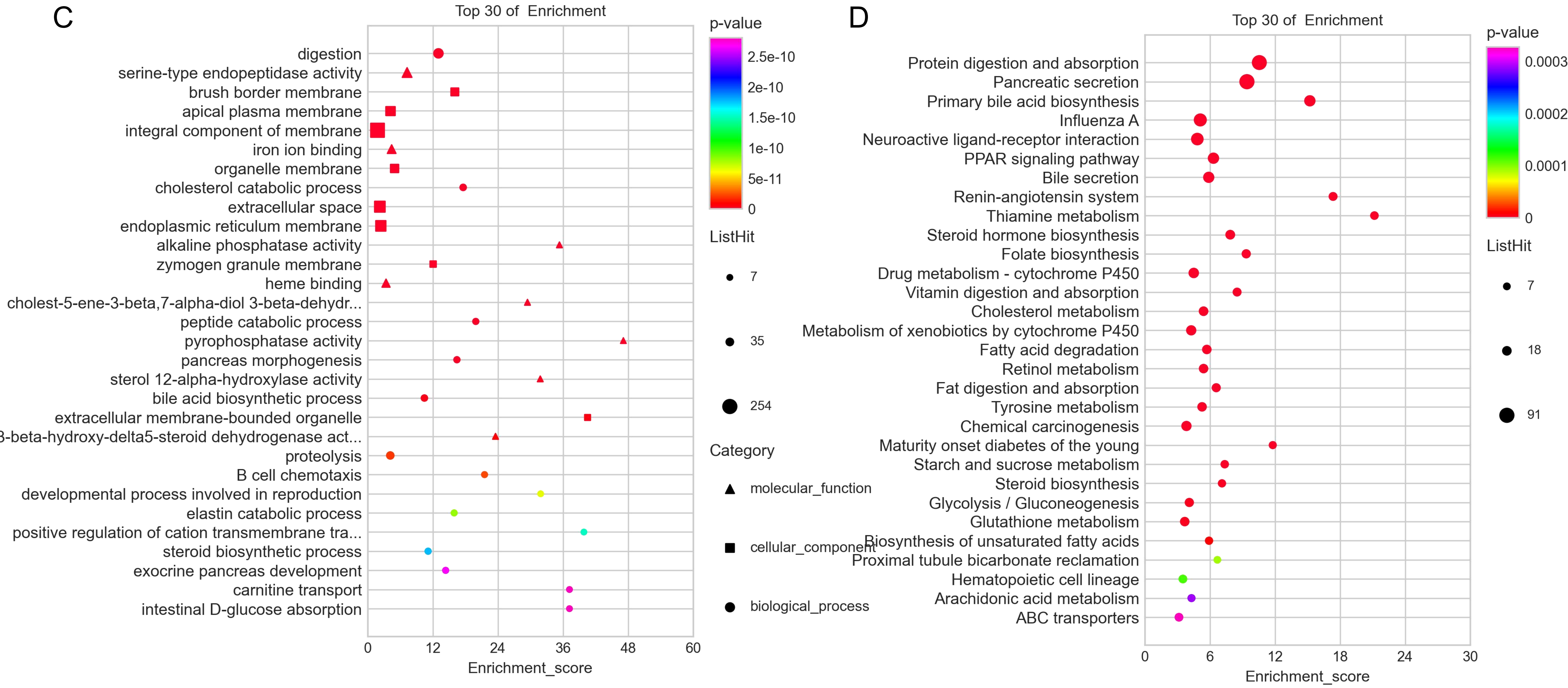

Fig. S5. Enrichment analysis of DEGs for *B. gargarizans* metamorphosis. The top 30 GO terms and pathways of significant enrichment for the (A and B) upregulated DEGs and (C and D) downregulated DEGs. The y-axis corresponds to the GO term or KEGG pathway, the x-axis corresponds to the EnrichmentScore, and the size of the point corresponds to the number of differential genes in the term or pathway.
