# Supplementary material for: Lipidomics combined with transcriptomic and mass spectrometry imaging analysis of the Asiatic toad (Bufo gargarizans) during metamorphosis and bufadienolide accumulation
Source: Chin Med. 2022 Nov 4;17:123. doi: 10.1186/s13020-022-00676-7 (PMC9636624; doi:10.1186/s13020-022-00676-7)
Supplement: Supplementary file 15 — Additional file 15: Fig. S8. Enrichment analysis of DEGs for BD accumulation. (A) By comparing G46 with G31, G38 and G42 respectively, 3260 common DEGs were identified. (B) and (C) showed the top 30 GO terms and pathways of significant enrichment of the BDs-related DEGs. [file 13020_2022_676_MOESM15_ESM.pdf]

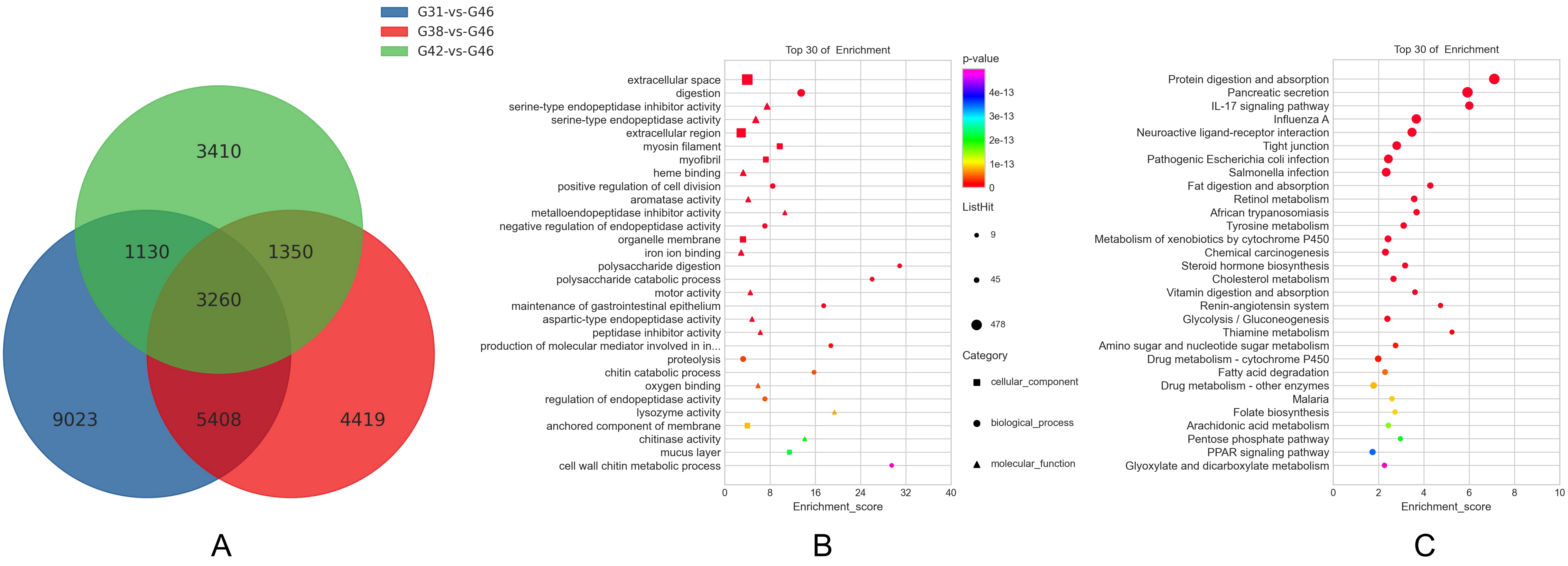

Fig. S8. Enrichment analysis of DEGs for BD accumulation. (A) By comparing G46 with G31, G38 and G42 respectively, 3260 common DEGs were identified. (B) and (C) showed the top 30 GO terms and pathways of significant enrichment of the BDs-related DEGs.
