# Supplementary material for: Lipidomics combined with transcriptomic and mass spectrometry imaging analysis of the Asiatic toad (Bufo gargarizans) during metamorphosis and bufadienolide accumulation
Source: Chin Med. 2022 Nov 4;17:123. doi: 10.1186/s13020-022-00676-7 (PMC9636624; doi:10.1186/s13020-022-00676-7)
Supplement: Supplementary file 16 — Additional file 16: Fig. S9. General overview of the major steps in DESI-MSI in the entire toad at the G46 stage. Step 1. After removing the limbs of the toads (G46 stage), whole-body cryosections were created along the sagittal and horizontal planes. Step 2. Serial tissue sections were collected while performing the optical microscopy and DESI-MSI techniques, and the appropriate sections were then selected for testing. DESI-MSI was performed, including generation of mass spectra and conversion to image, and identification of organs and tissues with optical microscopy. [file 13020_2022_676_MOESM16_ESM.pdf]

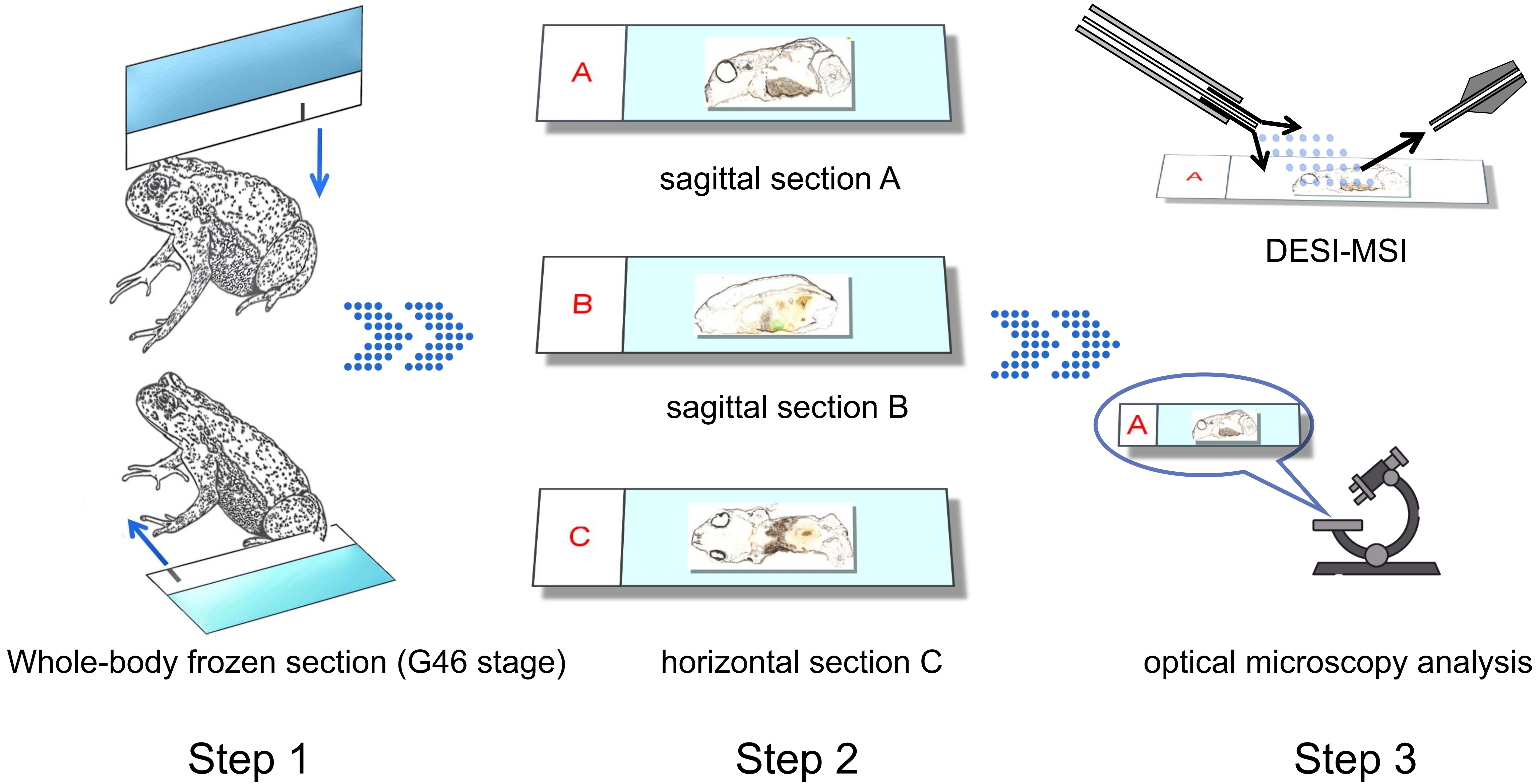

Fig. S9. General overview of the major steps in DESI-MSI in the entire toad at the G46 stage. Step 1. After removing the limbs of the toads (G46 stage), whole-body cryosections were created along the sagittal and horizontal planes. Step 2. Serial tissue sections were collected while performing the optical microscopy and DESI-MSI techniques, and the appropriate sections were then selected for testing. DESI-MSI was performed, including generation of mass spectra and conversion to image, and identification of organs and tissues with optical microscopy.
