# Supplementary material for: Lipidomics combined with transcriptomic and mass spectrometry imaging analysis of the Asiatic toad (Bufo gargarizans) during metamorphosis and bufadienolide accumulation
Source: Chin Med. 2022 Nov 4;17:123. doi: 10.1186/s13020-022-00676-7 (PMC9636624; doi:10.1186/s13020-022-00676-7)
Supplement: Supplementary file 17 — Additional file 17: Fig. S10. Identification of organs in DESI-MSI sections. (A–C) Frozen sections selected for DESI-MSI; (D–F) DESI-MSI at a specific mass-to-charge ratio that presents a clear org an position. (a–c) Microscope images of the corresponding parts of A to C. A, D and a representative of the left sagittal sections (sagittal section A); B, E, and b represent the mid-sagittal sections (sagittal section B); C, F, and c represent the horizontal sections (horizontal section C). [file 13020_2022_676_MOESM17_ESM.pdf]

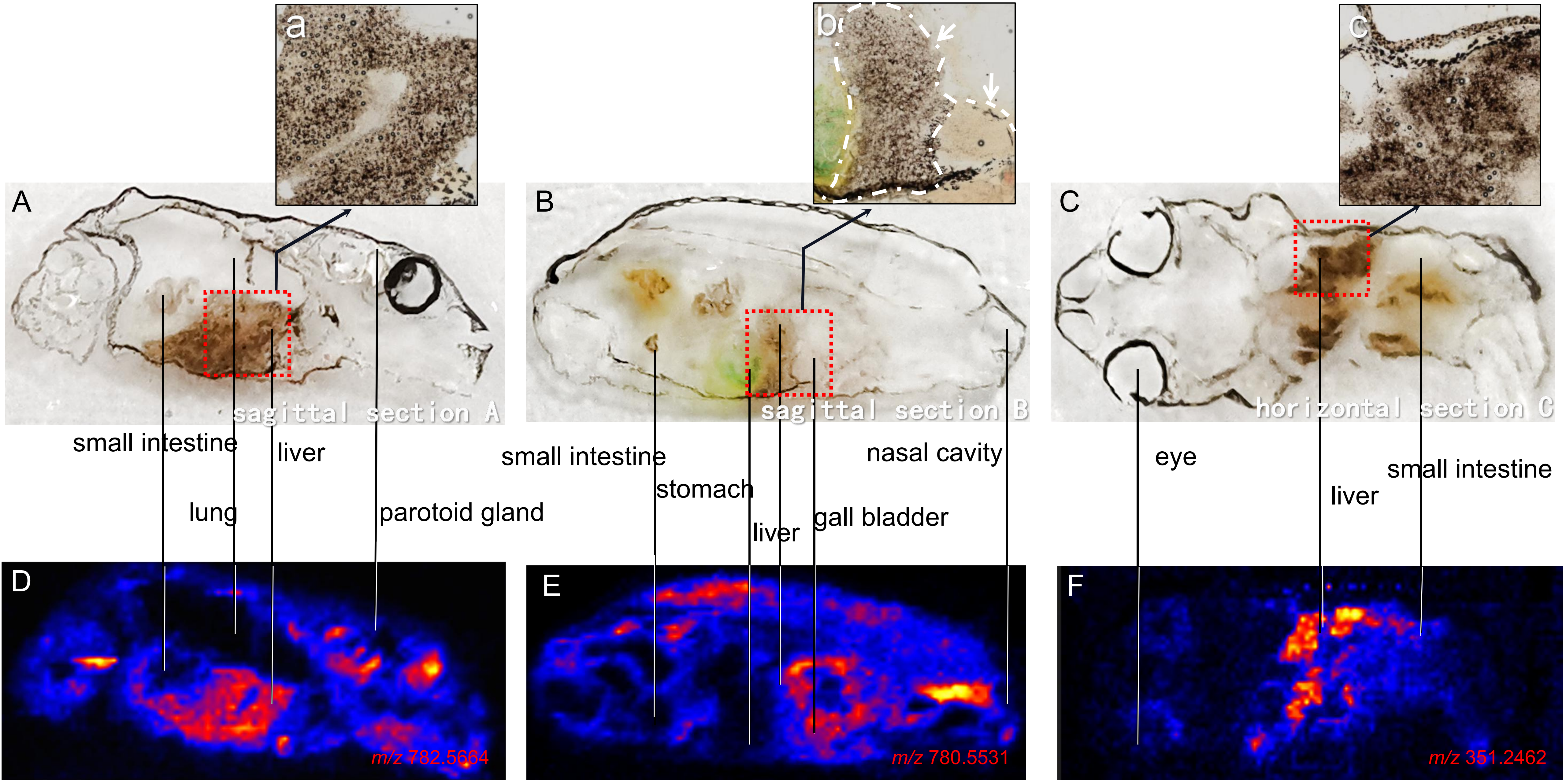

Fig. S10. Identification of organs in DESI-MSI sections. (A– C) Frozen sections selected for DESI-MSI; (D– F) DESI-MSI at a specific mass-to-charge ratio that presents a clear organ position. (a– c) Microscope images of the corresponding parts of A to C. A, D and a representative of the left sagittal sections (sagittal section A); B, E, and b represent the mid-sagittal sections (sagittal section B); C, F, and c represent the horizontal sections (horizontal section C).
